# Genomic insights in ascending aortic size and distensibility

**DOI:** 10.1016/j.ebiom.2021.103783

**Published:** 2021-12-28

**Authors:** Jan Walter Benjamins, Ming Wai Yeung, Yordi J. van de Vegte, M. Abdullah Said, Thijs van der Linden, Daan Ties, Luis E. Juarez-Orozco, Niek Verweij, Pim van der Harst

**Affiliations:** a University of Groningen, University Medical Center Groningen, Department of Cardiology, Groningen, the Netherlands; b Department of Heart and Lungs, University Medical Center Utrecht, University of Utrecht, Utrecht, the Netherland

**Keywords:** Ascending aorta size, Ascending aorta distensibility, Artificial intelligence, Cardiovascular disease, Genome-wide association study, Mendelian randomization study

## Abstract

**Background:**

Alterations in the anatomic and biomechanical properties of the ascending aorta (AAo) can give rise to various vascular pathologies. The aim of the current study is to gain additional insights in the biology of the AAo size and function.

**Methods:**

We developed an AI based analysis pipeline for the segmentation of the AAo, and the extraction of AAO parameters. We then performed genome-wide association studies of AAo maximum area, AAo minimum area and AAo distensibility in up to 37,910 individuals from the UK Biobank. Variants that were significantly associated with AAo phenotypes were used as instrumental variables in Mendelian randomization analyses to investigate potential causal relationships with coronary artery disease, myocardial infarction, stroke and aneurysms.

**Findings:**

Genome-wide association studies revealed a total of 107 SNPs in 78 loci. We annotated 101 candidate genes involved in various biological processes, including connective tissue development (*THSD4* and *COL6A3*). Mendelian randomization analyses showed a causal association with aneurysm development, but not with other vascular diseases.

**Interpretation:**

We identified 78 loci that provide insights into mechanisms underlying AAo size and function in the general population and provide genetic evidence for their role in aortic aneurysm development.

**Research in context**

**Search strategy**

We searched Google Scholar and PubMed for articles, published up to December 2020, related to aortic traits, genetic predisposition and the role of aortic traits in the development of cardiovascular disease, using the query ("aortic distensibility" OR "arterial stiffness") AND ("cardiovascular disease"[Title] OR "aortic aneurysm"[Title] OR "stroke"[Title] OR "myocardial infarction"[Title] OR "ischemia"[Title]). The search yielded 596 results, most of which are related to observational studies into blood pressure and arterial traits. These smaller-scale observational studies might be at risk of bias from confounding, reversed causation and collider bias.

**Evidence before this study**

- Multiple clinical /observational studies show that arterial stiffness and aortic distensibility are associated with aortic aneurysms and sudden death and can potentially be used for monitoring or early detection of cardiovascular diseases.
- There were limited studies in assessment of the utility of aortic biomarkers in large population-based cohorts.
- Previous genome wide association studies have revealed a limited number of loci associated with aortic traits and have been carried out in relatively small cohorts.

**Added value of this study**

- We developed a convolutional deep neural network to obtain aortic traits, namely ascending aortic cross-sectional area and ascending aortic distensibility, from contouring of cardiovascular magnetic resonance (CMR) data, which will be made available for download to facilitate future data-analyses and to fuel further research.
- We give an overview of ascending aortic traits in a large population-based cohort based on 3.8M cardiovascular CMR images from 37,910 individuals, which can be used as reference values for clinicians assessing aortic phenotypes.
- We find 107 genetic variants associated with AAo traits and identify 101 candidate genes, which increases our insights in AAo biology and might act as a starting point for drug development and precision medicine.
- Using a Mendelian randomization approach, we find a genetic association with aneurysms, highlighting the heritability of non-syndromic aneurysms in the general population. A genetic association with coronary artery disease, myocardial infarction, and stroke was not established.

**Implications of all the available evidence**

The results show that certain cardiovascular traits can easily be extracted from CMR-images using automated image assessment. Further research can focus on adding features to the algorithm in order to improve diagnosis and early detection of cardiovascular disease. In-vitro and in-vivo follow-up of the identified candidate genes for AAo size and function can be performed to validate their role and investigate possibilities for drug targeting. Randomized control trials will be necessary to confirm current Mendelian randomization analyses and to test whether treatment based on evaluation or targeting of AAo size and function improves cardiovascular disease outcomes.

Alt-text: Unlabelled box

## Introduction

The aorta is the largest elastic artery in the human body and functions as a conduit for blood pumped from the left ventricle as well as a dampener of the pulsatile pressure by distending and relaxing during systolic and diastolic phase, respectively.^1^^,^^2^ Alterations in the anatomic and biomechanical properties of the ascending aorta (AAo) can give rise to various vascular pathologies.

Anatomic properties of the AAo are essential for screening for AAo aneurysm and for AAo dissection, of which the latter is a major cause of sudden death.^3^^,^^4^ Genetic predisposition affects the risk of developing AAo aneurysm or dissection. Approximately 20% of aortic aneurysms is caused by single-gene disorders which show a highly penetrable familial pattern.^5^, ^6^, ^7^, ^8^ A heritable component to the remaining 80% of AAo aneurysms has also been suggested, but little is known about the predisp osing genetic factors in this phenotype and only a limited number of common variants has been identified so far.^9^^,^^10^

Biomechanical properties of the AAo, reflected by the distensibility or stiffness of the artery, are among the emerging biomarkers for monitoring vascular dysfunction, cardiovascular diseases and risk of all-cause mortality.^11^^,^^12^ However, it remains to be determined whether increasing AAo distensibility (AAo_dist_) translates into cardiovascular event reduction.^13^^,^^14^

New possibilities to directly capture AAo anatomy and distensibility have emerged due to the rise of large-scale studies collecting cardiac magnetic resonance (CMR) images and rapid advances of artificial intelligence (AI). The first aim of the present study is to perform segmentation of the AAo using our custom AI pipeline, and to generate an overview of normal values of AAo dimensional and functional features in 37,910 individuals in the UK Biobank.^15^^,^^16^ The second aim of the study is to expand current knowledge on the genetics of AAo anatomy and function by performing genome wide association studies (GWASs) and downstream functional annotation. The third and final aim is to investigate the potential causal relation between AAo traits and vascular disease development, including aneurysms, coronary artery disease and subtypes of stroke, using a Two-Sample Mendelian randomization (MR) approach.

## Methods

### Study population

**Figure 1:**
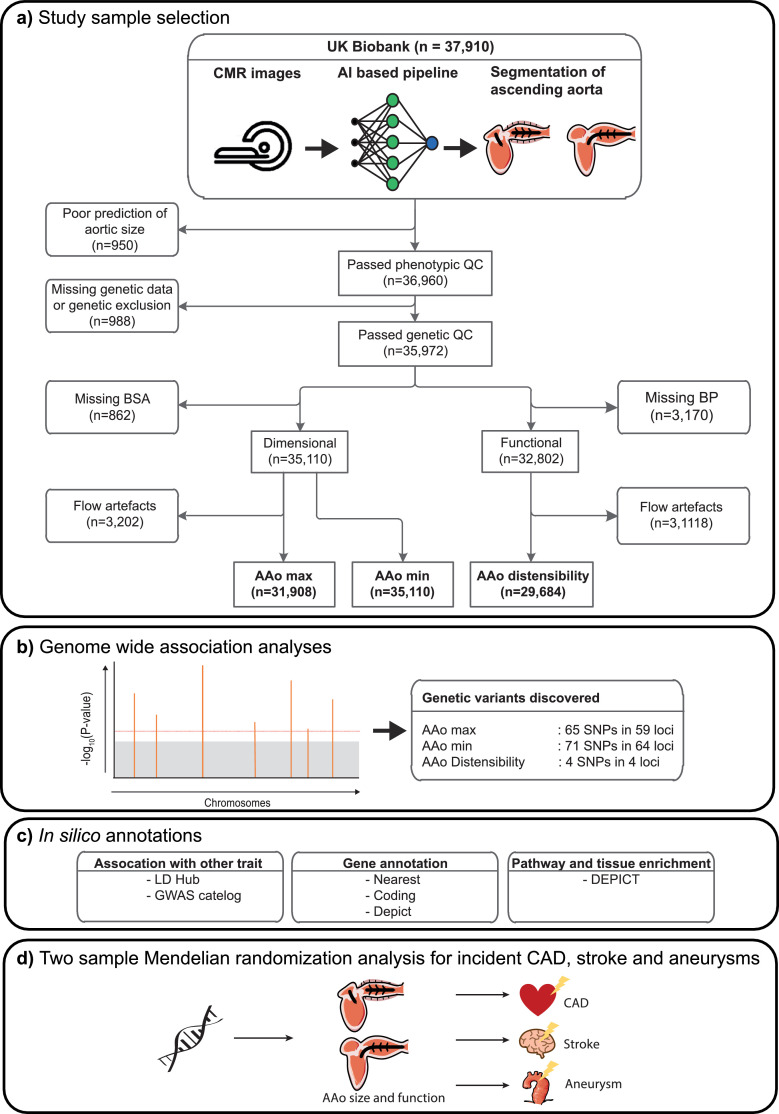
Study flowchart. Study flowchart depicting the exclusion criteria and number of individuals excluded for AAo_max_, AAo_min_ and AAo_dist_.

The UK Biobank study is a population-based cohort-study in which in total approximately 500,000 participants have been included. Further details on the UK Biobank study have previously been described in detail.^17^ A total of 37,910 participants who had undergone cardiac magnetic resonance (CMR) image acquisition were included in the current study. Figure 1 shows a flowchart of the study sample selection.

### Imaging data

MRI acquisition was scheduled during a separate imaging visit at which also other variables, such as blood pressure measurements, were acquired. Briefly, CMR scans of participants were acquired using a clinical wide bore 1·5 Tesla scanner (MAGNETOM Aera, Syngo Platform VD13A, Siemens Healthcare, Erlangen, Germany) in the absence of a pharmacological stressor or contrast agent. To derive aortic distensibility, a loop of 100 cine images was acquired during a single cardiac cycle for each participant. The images were acquired as a cardiac cine in a transverse cut at the level of the pulmonary trunk and the right pulmonary artery. An example of a CMR image with a corresponding annotation as it was predicted by the AI pipeline is displayed in *Supplementary Fig. 1*. Further details on the CMR protocol of the UK Biobank have previously been described.^18^ Images were made available as sets of DICOM files, each single cine-frame stored as a separate file.

### Image analysis

#### Model training

For 37,910 participants from the UK Biobank, the available imaging data was downloaded. PyDicom python library (v1.2.2) was used to extract pixel data from the source DICOM files and store the images as portable network graphics (PNG) files. NOUS (Cosmonio software, Leeuwarden, the Netherlands) active learning software was used to make an optimal selection of informative data for training a model to segment the ascending aorta (AAo). Because of technical limitations on the maximum number of images to be used within the software, a subset was pseudo-randomly selected from all images. From a subsample of 12,325 participants, two random frames out of the loop of 100 images per participant were selected. NOUS is an active learning software tool that allows to train DL models on a minimal set of ground truth images and provides the user with a set of images that optimally helps to train the currently developed model. A subset of 464 images was selected by the software for optimal training of the model and segmented manually inside the software by a human observer.

The set of 464 images segmentations was exported from NOUS to PNG files, and then used outside the active learning software, to train an improved U-Net model using Pytorch machine learning platform (v1.2.0).^19^ The images were randomly divided into a training set of 369 images, a validation set of 71 images and test set of 24 images. Model parameters were randomly initialized and optimized during training through back-propagation using an adaptive stochastic gradient based optimization algorithm called *Adam* with a training schedule to gradually reduce learning rate when learning performance stopped improving.^20^ To improve model robustness, and to avoid overfitting of the model, translation, resizing, and rotation were applied as image augmentation techniques during training. Random translations in both horizontal and vertical directions were limited to +/-0.0625 of image width and height, random scaling was limited between 0.8 and 1.2 of the input data, random rotation between -45° and 45°. The model was trained on batches of data from the training set iteratively, and the model with the best performance in the validation set was selected. The mean performance of the selected model on the test set, measured by the f1 score, was 0.967 (SD = 0.015).

#### Post processing – feature extractions

Open-CV computer vision library (v3.4.3.18) was used to extract contours from the binary prediction masks, which was then used to obtain the contour perimeter and the surface in pixel space. Also, from the contour data, the smallest bounding ellipse was obtained, using Open-CV. Two diameter values were obtained from the bounding ellipse. We calculated the average of the two diameters in pixel space to calculate a single AAo diameter. The Pixel Spacing DICOM tag was obtained from the original DICOM file using PyDicom python library (v1.2.2), to convert extracted values from pixel space to metric units, by multiplication of horizontal and vertical pixel dimensions with their respective pixel space values (mm/pixel).

We extracted AAo anatomy represented by cross-sectional area of the lumen during systole (AAo_max_) and diastole (AAo_min_) by converting the squared pixel areas to squared millimetres, using the DICOM Pixel spacing.

#### Post processing – quality control steps

From perimeter and surface, a relative roundness R_r_ of the contour was calculated, as described in Eq. (1), which can have a result value between 0, for an almost flat ellipse, and 1, for a perfect circle:

(1) $$R_{r}=4\pi *\frac{area}{perimeter^{2}}$$

Inter pixel variance σ_n_ was calculated as proposed by Immerkær using Eq. (2).^22^

(2) $$\sigma_{n}=\sqrt{\frac{\pi }{2}}\left(\frac{1}{6\left(W-2\right)\left(H-2\right)}\right)\Sigma_{Image\;I}\left|I_{\left(x,\;y\right)}*N\right|$$

Where *I* is the collection of all brightness values in the image, *W* is the image width, *H* is the image height and *N* is the noise estimation matrix (Eq. (3)), used in the convolution each pixel and the eight pixels surrounding it.

We witnessed high inter pixel brightness variances in images with flow void artefacts, a type of motion artefact caused by blood flow that results in blurring of the vessel wall. We therefore investigated the option to use the average interpixel variance for selection of images containing this type of artefacts. To focus on high interpixel variance around the ascending aorta and the heart, an 80 × 80 centred crop of the image was used to determine inter pixel variance.

(3) $$N=\left[\begin{array}{ccc} 1 & -2 & 1\\ -2 & 4 & -2\\ 1 & -2 & 1 \end{array}\right]$$

QC was performed after extraction of features from predicted segmentations of AAo. Thresholds for all criteria were determined after manual inspection of 7568 images by a panel of four human observers. Further details about exclusions have been described under Statistical analyses.

#### Automated pipeline

We implemented a fully automated pipeline, consisting of all aforementioned components, being the trained 2D convolutional neural network for segmentation of the AAo lumen, multiple quality QC steps to assess image and segmentation quality, and post-processing feature extractions, allowing the automated image analysis of 3.8M images from 37,900 individuals. The imaging pipeline ran on a set of multiple machines, each responsible for loading and analysis of a part of the total set of images, and for an update of the shared analysis results.

#### Model validation

A set of 50 participants was selected for validation of the model, independently and randomly, from the 25,586 studies whose images were not selected to train the model. From the selected studies, each tenth frame (frame 1, 11, 21, etc.) from the loop of 100 frames were segmented manually by two independent trained medical professionals using approved medical imaging software (CVI42, Circle Cardiovascular Imaging, Calgary, Alberta, Canada),). The same frames were exported from CVI42 to obtain images with the exact same zoom and pixel offset, to be automatically segmented by the model.

A total of 6 of the 50 selected studies were completely removed, because one or both human observers did not contour the aorta in the study due to poor image quality. One additional image from a single study was also removed because of poor image quality, resulting in 439 images from 44 studies, to be used for model validation.

Model performance was then estimated by the Sørensen-Dice similarity coefficient, also known as Dice coefficient or Dice metric (Eq. (4)), which divides twice the intersection of ground truth and prediction – in this data being manual and predicted contours in pixel-data – by the union of ground truth and prediction. A threshold of 0.90 was used for acceptance of the model.

(4) $$DSC=\frac{2\left|X\cap Y\right|}{\left|X\right|+\left|Y\right|}$$

To assess the similarity between features extracted from manual contours, as reported by CVI42, and features extracted by the post-processing pipeline described above, we chose to use the intra-class correlation coefficient (ICC). ICC was calculated between model and each observer, as well as between the two observers. A good (0.75–0.90) or excellent (> 0.90) ICC with two-way random-effects model was used for acceptance of values extracted from the model predictions.^23^

#### Data preparation

The extracted diameter and area values were indexed to body surface area (BSA) to correct for overall body size before use in genetic analyses.^21^ AAo_dist_ (in 10^−3^ mmHg^−1^) was calculated in participants with registered blood pressure measurements during the imaging visit, as the relative enlargement of the aorta during the systolic phase, divided by the pulse pressure (PP), as defined in Eq. (5):

(5) $$AAo\;distensibility=\;\frac{AAo_{max}-AAo_{min}}{AAo_{min}*PP}$$

Finally, correlation between traits was estimated using STATA 15 (StataCorp LP).

### Genotyping and imputation

Genetic data of the participants were genotyped using custom arrays, Affymetrix UK BiLEVE Axiom array or Affymetrix UK Biobank Axiom array which share 95% of marker content. The genotyping methods, arrays and quality-control procedures have been extensively described previously.^24^ SNPs with a minor allele frequency smaller than 0·5% or an INFO-score smaller than 0·3 were excluded from the association analysis.

### Statistical analyses

The sample size for training the deep learning model was determined on active learning software, as described under Image Analysis. All image data available from UK Biobank at initiation of the study were used to acquire the best representation of AAo size and function estimates from the population and were taken forward in the GWAS. Mendelian randomization analyses were performed in all non-related individuals from UK Biobank with available genetic data that were not included in the GWAS.

#### Exclusion criteria

After manual inspection of 7568 images with the corresponding predicted segmentations and extracted features by a panel of four human observers, we defined predictions to be unreliable at the image-level when the relative roundness R_r_ of the contour was below 0.85, or when AAo area was below 3 cm^2^, or above 20 cm^2^ which indicates a sub-optimal, non-perpendicular cross-sectional image acquisition of the ascending aorta, or the prediction of a physiological lumen or cavity other than the aorta. Furthermore, we decided to exclude all 100 frames from a single participant from subsequent analyses, when more than 25%–25 out of 100 frames – was excluded within that participant, or when the mean frame-to-frame difference between contour areas within one participant were above 0.3 cm^2^, which showed to be an additional measure for independent AAo lumen segmentations. Participants were excluded for analyses of AAo maximum area (AAo_max_) and derived AAo distensibility (AAo_dist_), when one or more frames yielded an inter pixel variance σ_n_ above 4.1. Since flow void artefacts mainly appear during systolic phase, we decided, not to use the appearance of flow void artefacts as exclusion criterion for analysis of AAo minimum area (AAo_min_). Additionally, for AAo_max_ and AAo_min_, additional individuals were excluded when BSA was missing. Finally, individuals were excluded for AAo_dist_, when blood pressure values were missing.

#### GWAS

Genome-wide association analyses were performed on inverse rank normalized values of AAo_max_, AAo_min_ and AAo_dist_, using BOLT-LMM (version: 2.3beta2), employing a mixed linear model which can take into account several potential sources of bias such as population structure and cryptic relatedness.^25^ All three AAo traits were corrected for age, sex, the first 30 principal components and the genotyping array. A total of 988 individuals were excluded based on failed genetic QC, defined by high heterozygosity and missingness as indicated by the Welcome Trust Centre for Human Genetics, and based on gender discrepancies between the reported and inferred gender. Linkage disequilibrium (LD)-based clumping was performed for each AAo trait to obtain independent SNPs using linkage disequilibrium (LD) cut-off of *R*^2^  < 0·005 within a five megabase window on variants passing the *P-*value threshold of 1 ×  10^−5^ using PLINK 1.9.^26^ To adjust for multiple testing, we applied Bonferroni correction using the genome-wide significance threshold of *P* < 5 × 10^−8^ for selection of independent SNPs that were considered significantly associated with AAo anatomy and distensibility. We scanned in a one megabase region at either side of the independent variant to define a genetic locus. Locus determination was repeated between AAo traits to obtain the strongest set of credible loci for the AAo traits combined (*Online* Table 1). Manhattan plots and QQ plots were generated using R V3.4.4. Genomic inflation was assessed by calculating lambda, LD score regression intercepts and attenuation ratios using LD score regression analyses.^27^^,^^28^ SNP heritability of AAo traits and the correlation between the traits were estimated using BOLT-REML.^29^

#### Embedment in previous GWAS results

The LD Hub platform was used to test for genetic correlations with previously performed GWAS.^27^^,^^30^ A Bonferroni-corrected significance of *P* < 0·05/855=5·85 × 10^−5^ was adopted to determine whether genetic correlations were significant. In addition, we queried the GWAS Catalog to find established genetic variants in linkage disequilibrium (LD) (*R*^2^ > 0·1) with AAo SNPs.^31^ GWAS Catalog summary statistics were downloaded from the NHGRI-EBI on 09/15/2020.^31^

#### Candidate causal genes

Three methods were applied to search for candidate causal genes for each AAo trait. First, we searched for the nearest gene or any gene within a 10kb distance of the lead SNP. Secondly, we searched for coding variants in LD (*R^2^* > 0·8) with the lead SNPs. Thirdly, we used DEPICT (DEPICT.v1.beta version rel137, obtained from https://data.broadinstitute.org/mpg/depict/) to identify candidate causal genes whilst taking gene-gene similarities and LD structures between loci into consideration.^32^ To identify variants with potential pathways to pathogenicity, we selected all lead SNPs for which coding variants in high LD were found with a CADD Phred score higher than 20. In the studied phenotypes in which the concerned coding variants were found, the identified variants were found in the top 1% of most deleterious variants. We then used these SNPs to compare prevalence of variants in individuals with or without coronary artery disease, any stroke, myocardial infarction, stroke, and aneurysms, to investigate potential pathogenicity, using a chi-square test.

#### Pathway and tissue enrichment analysis

DEPICT was also used to gain insights in enriched gene sets and tissues in which these genes are highly expressed.^32^ All lead SNPs at a *P*-value threshold of *P* < 1 × 10^−5^ were used to perform these analyses per AAo trait.

#### Mendelian randomization analysis

We aimed to investigate the potential causal relations between AAo traits and vascular diseases, including coronary artery disease (CAD), myocardial infarction, stroke and aneurysms. As exposure, we used all lead SNPs at a *P*-value threshold of *P* < 5 × 10^−8^ as instrumental variables per AAo trait.

### Outcomes in UK Biobank

Disease outcomes were captured based on a composite source of data from interview with a trained nurse at the visit to assessment centres (self-reported) and linked electronic health records including hospital inpatient episode data. Hospital inpatient episode data was collected at the Assessment Centre in-patient Health Episode Statistics (HES) in combination with data on cause of death from the National Health Service (NHS) Information Centre. HES and NHS data were respectively up to 31-03-2017 and 31-01-2018 for English participants, up to 29-02-2016 and 31-01-2018 for Welsh participants and up to 31-10-2016 and 30-11-2016 for Scottish participants.

Outcomes were assessed in the participants of the UK Biobank for which no AAo CMR images were available. From the full cohort, we excluded those individuals that withdrew informed consent (*n* = 171), those for which CMR images were available (*n* = 37,910), those who failed genetic quality control (*n* = 1251) and exclusions based on familial relatedness (n = 82,008). A total of 381,324 unrelated individuals that were not in any of the GWAS discovery cohorts remained available to test the SNP-outcome associations. Outcomes included coronary artery disease, myocardial infarction, any stroke, ischemic stroke, haemorrhagic stroke and aneurysms. Disease definitions are further detailed in the Supplementary File 3, *Tables 11 and 12*. Logistic regressions were performed to obtain SNP-outcome associations on ever having received the diagnosis in an individual's lifetime. All regression analyses were corrected for age on the date of the last follow-up, sex, the first 30 principal components and the genotyping array. Regression analyses were performed using statistical software STATA 15 (StataCorp LP).

### Outcomes in external cohorts

Replication of the association between coronary artery disease and stroke was performed in the independent cohorts of the CARDIoGRAMplusC4D and MEGASTROKE consortia, for which detailed descriptions have been published previously.^33^^,^^34^ Additional information on the consortia can be found in *Online Table 13.* Unfortunately, summary statistics of GWAS on thoracic aneurysms were not available to us. Proxies were searched in the scenario that variants were not available in the summary statistics of the CARDIoGRAMplusC4D and MEGASTROKE consortia.^33^^,^^34^ Only high LD proxies (LD >0·8) were taken forward in further analyses.

### Data harmonization

Exposure and outcome summary statistics were harmonized using the TwoSample MR package.^35^ We used the recommended settings in which forward strand alleles are inferred using allele frequency information, but, where possible, palindromic SNPs were removed if the minor allele frequency was above 0·42.^35^

### Weak instrument bias

The first assumption of MR is that genetic variants should be reliably associated with the exposure. We used the *F*-statistic to test whether this assumption is statistically fulfilled.^36^ The *F*-statistic was calculated per genetic variant as followed: *F* *= ((R^2^ × (n-2))/(1-R^2^)*.^37^ Here, *n* is the exposure's sample size and *R*^2^ is the amount of variance the SNP explains of the exposure, calculated using a previously established formula.^37^^,^^38^ We used a cut-off of *F*-statistic > 10 as indication of absence of weak instrument bias.^36^

### Reverse causation

The third assumption of MR studies is that genetic variants are only associated with the outcome through the risk factor. One scenario in which this assumption is violated is when the genetic variant is stronger associated with the outcome, i.e. reverse causation. To prevent this, we applied MR-Steiger filtering.^39^ We assessed the R^2^ for the exposure and outcome and removed any genetic variants that were significantly stronger associated with the outcome than with the exposure per exposure-outcome association. R^2^ for linear and logistic traits were calculated based on the GWAS summary statistics according to previously established formulae.^38^^,^^40^

### Heterogeneity and sensitivity analyses

An inverse variance weighted random effects model (IVW-RE) was applied to estimate the causal effect of AAo size and function on all outcomes.^41^ The IVW model only holds a true causal estimate when the third assumption is satisfied, and all SNPs are valid. Inclusion of variants that are associated with the outcome through other risk factors than the assessed exposure, i.e. pleiotropic variants, would distort the causal estimates.^41^ We used the Rücker framework to gain insights in any pleiotropy within our MR estimates.^42^ Significant heterogeneity within the IVW estimate, as indicated by a significant Cochran's Q (*P <* 0·05) in combination with an I^2^ index > 25% was used as statistical evidence for at least balanced horizontal pleiotropy.^42^^,^^43^ We assumed at least some horizontal pleiotropy in every IVW estimate and erred on the conservative side by choosing an IVW random effects model for our main MR analysis. Following the Rücker framework, we performed the MR-Egger test which does restrain estimates to a zero intercept and therefore allows for exertion of unbalanced horizontal pleiotropic effects.^42^^,^^44^ A significant difference between the heterogeneity estimates within the IVW and MR-Egger model, i.e. a Q-Q' with a *P* < 0·05, in combination with a significant non-zero intercept of the MR-Egger regression (*P* < 0·05) was considered to indicate unbalanced horizontal pleiotropy.^42^ Under this scenario, we used the MR-Egger test for our main MR analysis.^44^ Weak instrument bias in the MR-Egger regression analysis was assessed by I^2^_GX_ and, with an I^2^_GX_ > 95%, indicating low risk of measurement error.^45^

There are also possible scenarios in which only a small or a large proportion of the included genetic variants exert pleiotropic effects and we therefore performed several sensitivity analyses. We used the MR-Lasso, MR-RAPS (both down-weight outliers) and MR-PRESSO approaches (removes outliers based on the residuals) to provide robust MR in the situation that a small proportion of the genetic variants exert pleiotropic effects.^46^, ^47^, ^48^ We used the weighted median (assumes the majority of the SNPs to be valid) and the weighted mode and MR-Mix approaches (assume the plurality of the SNPs to be valid) to provide robust MR in the situation that a large proportion of the genetic variants exert pleiotropic effects.^49^, ^50^, ^51^

MR analyses were performed using R (version 3.6.3), the TwoSampleMR package (version 0.5.3), MR-PRESSO package (version 1.0), MR-Lasso source code and the MR-mix package.^35^^,^^46^^,^^48^^,^^51^^,^^52^ A Bonferroni corrected *P*-value of *P* < 0·05/10 outcomes = 0·005 was considered to be significant for the main IVW random effects analysis. A *P-*value cut-off of *P* < 0·05 was considered to be suggestively significant for the main analysis and to indicate a significant association for the generally weaker powered sensitivity analysis.

### Ethics

All data provided by UK biobank was acquired after approval from North West - Haydock Research Ethics Committee (REC reference: 16/NW/0274). All participants of the UK biobank have provided written informed consent.

CARDIoGRAMplusC4D and MEGASTROKE consortia are ethically approved by local and institutional committees. For more information, see the original consortium papers.^33^^,^^34^

### Role of funding sources

In the present study, none of the funding sources played a role in study design, data collection, data analyses, interpretation, or writing the manuscript.

## Results

### Model validation results

The model yielded a Dice score of 0.93 (SD=0.03), compared to observer 1 and 0.95 (SD=0.02) compared to observer 2, thereby passing the desired threshold of 0.90, and we deemed the model's performance to be sufficient for assessment of aortic dimensions and function in the whole set of MRI images downloaded from the UK Biobank.

Model vs observer comparison of extracted areas yielded an ICC of 0.96 (*P* < 0.001, ICC with two-way random -effects model) in comparison to observer 1 and 0.95 (*P* < 0.001, ICC with two-way random -effects model) compared to observer 2, where a comparison between both human observers yielded an ICC of 0.99 (*P* < 0.001, ICC with two-way random -effects model). For distensibility the comparison between model and observer 1 yielded an ICC of 0.90 (*P* < 0.001, two-way random effects model), compared to observer 2 0.85 (*P* < 0.001, ICC with two-way random -effects model), between both observers 0.85 (*P* < 0.001, ICC with two-way random -effects model). Overall, the ICC values for distensibility were lower than for area. However, in contrast to area, we observed a higher ICC between model and both individual observers than the ICC between observers for distensibility.

### Exclusions

The U-Net model was deployed to segment the ascending aorta in 37,910 participants who underwent CMR imaging. After segmentation, a total of 950 participants were excluded on a per-phenotype basis, of which 440 exclusions were based on low roundness, 272 were excluded based on high area values, and 238 participants were excluded based on high average inter-frame area changes. No low values of AAo area were encountered after exclusion of predicted contours with a low roundness. Additionally, manual evaluation of values exceeding the low and high value thresholds revealed, that all these values were the results of model mispredictions, most of which occurred due to the presence of flow void artefacts in the source images.

During preparation of phenotype data, 988 individuals were dropped because of missing principal component values or information about the used genotyping chip. For AAo_max_ and AAo_min_, 862 participants were excluded that did not contain information on BSA. For AAo_dist_, 3170 participants were excluded because of missing blood pressure values. For AAo_max_, 3202 cases were dropped because of high inter-pixel brightness variances, indicating flow artefacts. 3118 cases with high inter-pixel brightness were dropped for AAo_dist_. A flowchart of the study sample selection can be found in Figure 1. After exclusion of these participants, we used estimated areas from the remaining 35,110 participants for subsequent analysis of AAo_min_, 31,908 for analysis of AAo_max_ and 29,684 for analysis of AAo_dist_.

### Observational correlations between aortic traits

We estimated correlation between all three traits, measured by Pearson's product-moment correlation coefficient. We calculated a correlation coefficient of 0·959 (CI 0·958–0·960, *P* < 0.001, Pearson's correlation) between AAo_max_ and AAo_min_. Correlations between both Area traits and AAo_dist_ were substantially lower. Correlation between AAo_max_ and AAo_dist_ yielded a value of -0·142 (CI -0·153–-0·131, *P* < 0.001, Pearson's correlation), versus correlation between AAo_min_ and AAo_dist_ of -0·365 (CI -0·375–-0·355, *P* < 0.001, Pearson's correlation).

### Population characteristics and normal values

Table 1 shows the characteristics of the study population for all individuals for which information on all three AAo traits was present after QC as well as per AAo trait. The mean age was 64·1 years with 48·4% being males. The mean BMI was 26·4 kg/m^2^ and 23·8% of the population had a history of hypertension. The mean AAo_max_ was 4·38 cm^2^/m^2^, mean AAo_min_ was 3·89 cm^2^/m^2^, and mean AAo_dist_ was 2·25 × 10^−3^ mmHg^−1^. Reference values of the aortic phenotypes, stratified by age groups and sex, can be found in Table 2 and *Supplementary Fig. 2*.

**Table 1:** Baseline characteristics.

Factor	All	AAo_max_	AAo_min_	AAo_dist_
Sample size	29,381	31,908	35,110	29,684
Sex (% female)	52.6	52.7	51.5	52.6
Age (years)	64.1 ± 7.5	64.20 ± 7.54	64.10 ± 7.54	64.12 ± 7.54
Systolic blood pressure (mmHg)	133.6 ± 17.8	133.6 ± 17.8	133.8 ± 17.8	133.6 ± 17.8
Diastolic blood pressure (mmHg)	78.9 ± 8.5	78.9 ± 8.5	79.1 ± 8.5	78.9 ± 8.5
Pulse pressure (mmHg)	54.6 ± 13.7	54.6 ± 13.7	54.7 ± 13.8	54.7 ± 13.8
Smoking behaviour (%)				
*Never or <100 cigarettes*	64.3	64.3	63.9	64.3
*Stopped <=12 months*	0.1	0.1	0.1	0.1
*Stopped >12 months*	31.9	32.0	32.4	32.0
*Active occasionally*	1.4	1.4	1.5	1.4
*Active daily*	2.2	2.2	2.2	2.2
BMI (kg/m^2^)	4.14	4.15	4.29	4.14
Hypertension (%)	23.2	23.2	23.8	23.2
Diabetes Mellitus (%)	2.9	2.9	3.2	2.9
Hyperlipidemia (%)	12.9	12.9	13.4	12.9
Coronary Artery Disease (%)	2.1	2.2	2.2	2.1
AAo_max_, mean (cm^2^)	8.1 ± 1.7	8.1 ± 1.7	8.1 ± 1.8	8.1 ± 1.7
AAo_max_ / BSA, mean (cm^2^/m^2^)	4.4 ± 0.9	4.4 ± 0.9	4.4 ± 0.9	4.4 ± 0.9
AAo_min_, mean (cm^2^)	7.2 ± 1.6	7.2 ± 1.6	7.2 ± 1.6	7.2 ± 1.6
AAo_min_ / BSA, mean (cm^2^/m^2^)	3.9 ± 0.8	3.9 ± 0.8	3.9 ± 0.8	3.9 ± 0.8
AAD, mean (10^−3^ mmHg^−1^)	2.3 ± 1.3	2.3 ± 1.3	2.3 ± 1.3	2.2 ± 1.3
BSA (m^2^)	1.9 ± 0.2	1.9 ± 0.2	1.9 ± 0.21	1.9 ± 0.2

*Baseline characteristics for those who had available data for all AAo traits, as well as those who had available data for AAo_max_, AAo_min_ and* AAo_dist_*. Continuous variables are presented as mean±SD and binary variables as percentages. AAo=ascending aorta; AAo_max_=ascending aorta size during systole; AAo_min_=ascending aorta size during diastole; AAo_dist_=ascending aorta distensibility; BSA=Body Surface area.*

**Table 2:** Normal values for AAo_max_, AAo_min_ and AAo_dist_, stratified by age and sex.

Phenotype	Sex	Age <55		Age 55–65		Age 65–75		Age>75	
			N		N		N		N
AAo_min_, mean (cm^2^)	All	6.46 (1.47)	5,467	7.00 (1.59)	13,550	7.53 (1.61)	15,436	7.75 (1.65)	2,507
	Men	7.04 (1.46)	2,463	7.66 (1.61)	6022	8.11 (1.61)	7,864	8.23 (1.64)	1,497
	Women	5.98 (1.30)	3,004	6.48 (1.36)	7528	6.93 (1.36)	7,572	7.05 (1.38)	1,010
AAo_min_ / BSA, mean (cm^2^/m^2^)	All	3.42 (0.71)	5,349	3.76 (0.78)	13,264	4.08 (0.81)	15,051	4.23 (0.84)	2,414
	Men	3.46 (0.69)	2,415	3.80 (0.77)	5908	4.11 (0.81)	7,680	4.26 (0.83)	1,452
	Women	3.39 (0.71)	2,934	3.73 (0.78)	7356	4.06 (0.80)	7,371	4.19 (0.85)	962
AAo_max_, mean (cm^2^)	All	7.55 (1.59)	4,854	7.91 (1.70)	12,254	8.37 (1.75)	14,130	8.59 (1.81)	2,366
	Men	8.18 (1.59)	2,121	8.68 (1.71)	5286	9.07 (1.73)	7,029	9.17 (1.78)	1,391
	Women	7.05 (1.41)	2,733	7.33 (1.44)	6968	7.67 (1.45)	7,101	7.77 (1.52)	975
AAo_max_ / BSA, mean (cm^2^/m^2^)	All	4.01 (0.77)	4,747	4.27 (0.82)	11,999	4.55 (0.87)	13,768	4.70 (0.92)	2,277
	Men	4.04 (0.76)	2,077	4.32 (0.82)	5188	4.60 (0.87)	6,858	4.76 (0.91)	1,348
	Women	3.99 (0.77)	2,670	4.23 (0.82)	6811	4.49 (0.85)	6,910	4.61 (0.92)	929
AAo_dist_, mean (10^−3^ mmHg^−1^)	All	3.34 (1.41)	4,503	2.45 (1.21)	11,143	1.79 (0.97)	12,812	1.55 (0.96)	2,039
	Men	2.86 (1.00)	1,976	2.28 (1.11)	4817	1.77 (0.99)	6,400	1.58 (1.03)	1,210
	Women	3.72 (1.57)	2,527	2.58 (1.27)	6326	1.81 (0.95)	6,412	1.52 (0.85)	829
AAo Diam_min_, mean (mm)	All	17.34 (2.00)	5,467	18.09 (2.09)	13,550	18.80 (2.05)	15,436	19.09 (2.07)	2,507
	Men	18.15 (1.91)	2,463	18.96 (2.04)	6022	19.55 (1.99)	7,864	19.70 (2.01)	1,497
	Women	16.68 (1.82)	3,004	17.39 (1.86)	7528	18.02 (1.80)	7,572	18.19 (1.81)	1,010
AAo Diam._min_ / BSA, mean (mm/m^2^)	All	9.23 (1.12)	5,349	9.76 (1.20)	13,264	10.23 (1.22)	15,051	10.46 (1.24)	2,414
	Men	9.71 (1.05)	2,415	10.10 (1.10)	5908	10.52 (1.14)	7,680	10.84 (1.17)	1,452
	Women	9.47 (1.15)	2,934	10.03 (1.22)	7356	10.57 (1.23)	7,371	10.82 (1.30)	962
AAo Diam._max_, mean (mm)	All	18.86 (2.00)	4,854	19.33 (2.09)	12,254	19.89 (2.09)	14,130	20.17 (2.13)	2,366
	Men	19.67 (1.92)	2,121	20.28 (2.02)	5286	20.74 (2.01)	7,029	20.87 (2.03)	1,391
	Women	18.24 (1.83)	2,733	18.61 (1.83)	6968	19.05 (1.81)	7,101	19.16 (1.85)	975
AAo Diam._max_ / BSA, mean (mm/m^2^)	All	10.09 (1.16)	4,747	10.48 (1.21)	11,999	10.87 (1.24)	13,768	11.07 (1.26)	2,277
	Men	9.73 (1.05)	2,077	10.12 (1.10)	5188	10.55 (1.15)	6,858	10.85 (1.16)	1,348
	Women	10.36 (1.16)	2,670	10.75 (1.21)	6,811	11.18 (1.24)	6,910	11.40 (1.31)	929

### Genome-wide analyses of ascending aorta traits

**Figure 2:**
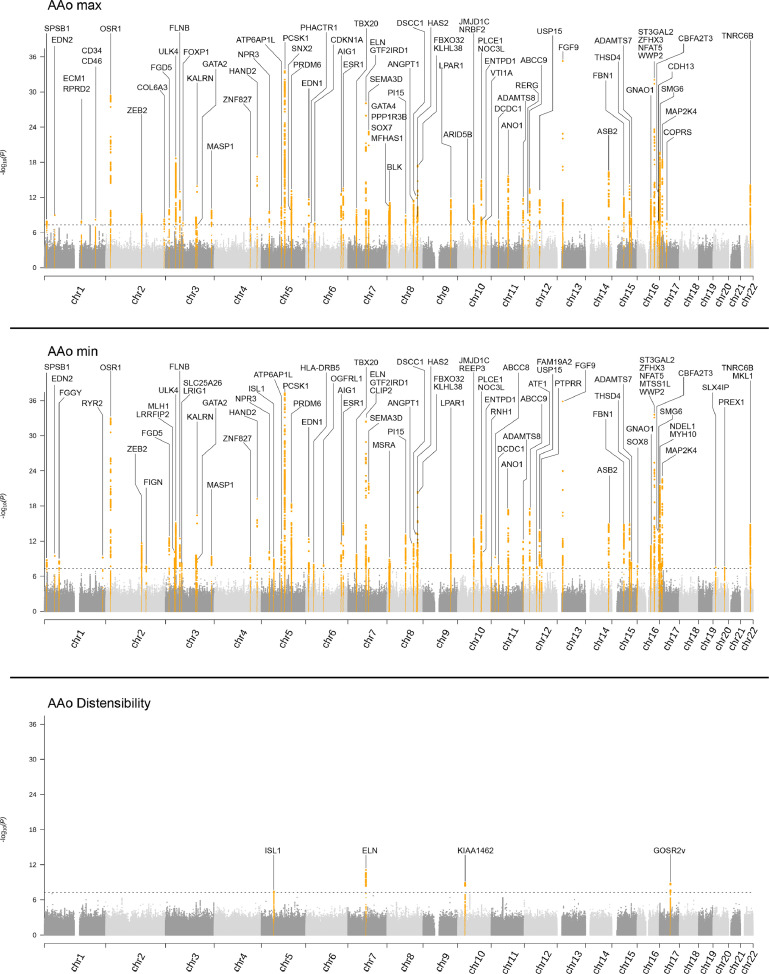
Manhattan plot shows the results for the GWAS's of AAo anatomy and function. The Manhattan plots shows the results of the GWSA of A) AAo_max_, B) AAo_min_ and C) AAo_dist_. Loci reaching genome-wide significance (*P* < 5 × 10^−8^) are coloured red and annotated with the identified genes. The *Y*-axis shows the –log_10_(*P*-value), the *X*-axis chromosome 1–22.

GWAS revealed a total of 107 variants in 78 loci across all three AAo traits (Supplementary 3, *Table 1*). We found 65 SNPs in 59 loci for AAo_max_, 71 SNPs in 64 loci for AAo_min_ and 4 SNPs in 4 loci for AAo_dist_ (Figure 2). In line with the high observational correlation between the phenotypes, we found 47 out of 76 loci shared between AAo_max_ and AAo_min_. In addition, AAo_max_ and AAo_min_ showed an almost perfect genetic correlations (r_g_=0·994 ± 0·002, LD score regression), while their respective correlation with AAo_dist_ was lower (r_g_=-0·307 ± 0·054; r_g_=-0·381 ± 0·047, LD score regression). BOLT-REML estimated high SNP-based heritability for both AAo_max_ (h^2^_g_=0·509 ± 0·019, Monte Carlo variance components analysis) and AAo_min_ (h^2^_g_=0·502 ± 0·019, Monte Carlo variance components analysis), but a lower heritability for AAo_dist_ (h^2^_g_=0·151 ± 0·020, Monte Carlo variance components analysis). The identified variants explained 10·2% variance of AAo_max_, 10·7% for AAo_min_ and 0·7% for AAo_dist_.

### Embedment in previous genetic studies

We found that the SNPs identified in the current study were in high LD (*R*^2^  > 0·8) with previously established variants for aortic size or thoracic aneurysms (four SNPs), blood pressure traits (12 SNPs) and atherosclerosis and thrombosis (two SNPs) (*Online Table 2*). LDhub was queried to assess genetic correlations with other traits and showed that AAo traits were mainly correlated with blood pressure and anthropometric traits (*Online Table 3*).

### Candidate causal genes

We searched for candidate causal genes using multiple strategies and identified a total of 101 genes for which we provide a framework for their possible involvement in AAo anatomy and function in Figure 4. We identified 72 genes for AAomax,(72 genes) 81 for AAomin and four for AAodist. The genes identified for AAomax and AAomin showed significant overlap with 53 shared genes. All three methods prioritized *JMJD1C* and *ADAMTS7* (Figure 4). ELN was shared between all AAo traits (*Supplementary Fig. 5*). Annotation and further information of all identified genes is provided in Online Table 6. FBN1, ELN, MYH10 and ULK4 showed overlap with genes that have previously been associated with syndromic, familial and sporadic aneurysms (Supplementary Table 1, Online Fig. 5).^53^, ^54^, ^55^, ^56^, ^57^, ^58^, ^59^ A total of six genetic variants was in high LD with coding variants of *JMJD1C, SMG6, NOC3L, ULK4*, and *ADAMTS7*. We assessed the likelihood of their pathogenicity according to the American College of Medical Genetics (ACMG) guidelines. There was only evidence for differences in the prevalence of one variant in *SMG6* between case and control groups for coronary artery disease in the UK Biobank (*Online Table 13*). The relative CADD scores of coding variants of *ULK4* (rs3774372, in high LD with rs60248638) and *SMG6* (rs903160, in high LD with indel 17:2088848_CCAGA_C) were higher than 20 and therefore belong to the top 1% of most pathogenic coding variants.

### Pathway and tissue enrichment analysis

Pathway enrichment analysis revealed 223 reconstituted gene sets in 30 gene clusters for AAo_max_, 419 gene sets in 53 gene clusters for AAo_min_, but none for AAo_dist_. 178 gene sets were significantly enriched for both AAo_max_ and AAo_min_. The central nodes of the top gene clusters for AAo_max_ were complete embryonic lethality during organogenesis, response to nutrient levels and circulatory system processes, for AAo_min_ the CREBBP PPI subnetwork, smooth muscle cell proliferation and anemia. (*Online Table 7*). Tissue enrichment analysis revealed six significantly associated tissues for AAo_max_ and 10 significantly associated tissues for AAo_min_. We found no significantly enriched tissues for AAo_dist_. Both AAo_max_ and AAo_min_ were strongest enriched in the arteries and all top five tissues were related to cardiovascular system. Full results of the tissue enrichment analysis can be found in *Online Table 8* and are shown in *Supplementary Fig. 4*.

### MR analysis

A series of Two-sample MR analyses was performed to investigate the potential causal mechanisms between AAo size and function and cardiovascular diseases. Results of the heterogeneity and sensitivity analyses are described in more details in the supplement and in *Online Tables 9* and *10*.

**Figure 3:**
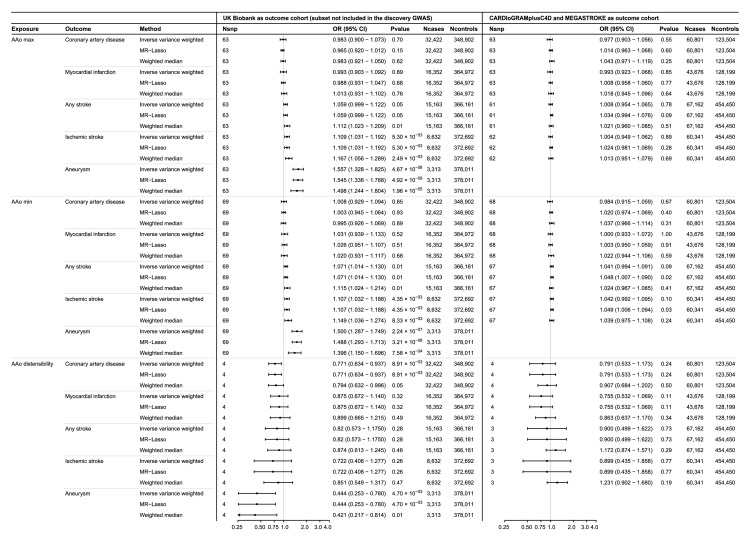
Forestplot of the two-sample Mendelian randomization (MR) estimates of AAo anatomy and function on vascular diseases. Forestplot of the two-sample Mendelian randomization (MR) estimates of AAo_max_, AAo_min_ and AAo_dist_ on coronary artery disease, myocardial infarction, stroke, ischemic stroke and aneurysm development. The outcomes were assessed in an independent subset of individuals in the UK Biobank (left panel) and in CARDIoGRAMplusC4D and MEGASTROKE consortia (right panel). We show the effect sizes of the main inverse variance weighted random effect model, as well as the MR-Lasso method and weighted median method (which are robust to the scenario in which respectively a small proportion or up to half of the genetic variants to exert pleiotropic effects). A Bonferroni corrected *P*-value of *P* < 0•05/10 outcomes=0•005 was considered significant for the main inverse variance weighted random effects analysis. A *P-*value cut-off of *P* < 0•05 was considered to be significant for sensitivity analysis. The *X*-axis show the odds ratios and 95% confidence intervals. OR odds ratio, CI confidence interval.

We found no evidence for a genetic association between AAo_max_, AAo_min_, or AAo_dist_ and coronary artery disease or myocardial infarction in the UK Biobank or in the CARDIoGRAMplusC4D cohort using an inverse variance weighted random-effects model (Figure 3). AAo_max_ and AAo_min_ were genetically associated with ischemic stroke at a suggestive *P*-value threshold of 0.05 (OR 1·109, CI 1·031–1·192, *P* = 5·30 × 10^−3^; OR 1·107, CI 1·032–1·188, *P* = 4·35 × 10^−3^, respectively, IVW-RE)., but the results were not replicated in the MEGASTROKE consortium for both AAo_max_ (OR 1·004, CI 0·949–1·062, *P* = 8·93 × 10^−1^, IVW-RE) and AAo_min_ (OR 1·042, CI 0·992–1·095, *P* = 1·02 × 10^−1^, IVW-RE) (Figure 3). We also found a suggestively significant association between AAo_min_ and any stroke in the UK Biobank, but again this result was not replicated. AAo_dist_ was not associated with any or ischemic stroke. We did not find a genetic association between AAo anatomy and AAo distensibility and other stroke subtypes, including subarachnoidal haemorrhage, intracerebral haemorrhage, cardio-embolic, large-artery and small-vessel stroke (*Online Table 10*).

**Figure 4:**
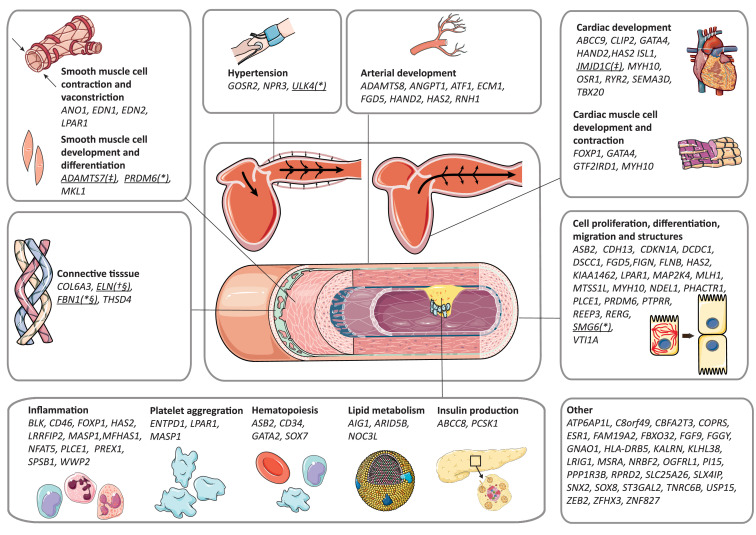
Conceptual biological framework of 101 genes implicated in AAo size and function. Biological framework of the candidate genes for AAo size and function as identified by the nearest, coding, DEPICT or a combination of these methods. Further information on gene function can be found in Online Table 6 and a list of genes that were previously implicated in AAo size, function or thoracic aneurysm development in Supplementary Table 1. (*) in the same region as previously identified genes for AAo size or sporadic thoracic aneurysms, (§) in the same region as previously identified genes known to cause syndromic thoracic aneurysms, (†) identified for AAo_max_, AAo_min_ and AAo_dist_, (‡) identified by all gene prioritization methods.

Lastly, we studied the association between AAo size and function and aneurysms. We found strong evidence for a genetic association between AAo_max_, AAo_min_, AAo_dist_ and aneurysms (OR 1·557, CI 1·328–1·825, *P* = 4·67 × 10^−8^; OR 1·500, CI 1·287–1·748, *P* = 2·24 × 10^−7^; 0·453, CI 0·243–0·780, *P* = 4·70 × 10^−3^, respectively, IVW-RE). The results were robust to MR-Lasso and weighted median analyses (Figure 3). Considering the limited number of genetic variants associated with AAo_dist_, we lowered the *P-*value threshold for inclusion of genetic variants to *P* < 1 × 10^−6^ and found genetic associations generally consistent with the main results for all exposure-outcome associations (*Online Table 10*).

## Discussion

In the current study, we applied automated segmentation of the AAo lumen using an AI approach, to obtain its properties on 37,910 participants from the UK Biobank. GWAS from these derived phenotypes identified a total of 107 common genetic variants in 78 loci associated with AAo anatomy and function and *in silico* annotations provide insights in their biological background. The MR analyses provide evidence for a genetic association between AAo anatomy and development of aneurysms in a general population.

We provide normal values of AAo anatomy and function in the largest cross-sectional population-based cohort study to date. We additionally characterized age-related changes in the aorta for women and men and show that there is an age-dependent increase of AAo size and decline of AAo_dist_. The average AAo_dist_ was higher for women than men younger than 65 years, but this effect was attenuated within the older age categories. The reference values were similar to previous studies and provide a framework for physicians to evaluate AAo anatomy from CMR imaging.^15^^,^^60^, ^61^, ^62^

We then aimed to gain additional insights in the biology of interindividual differences in AAo size and identified a total of 103 common genome-wide significant genetic variants. There was a high overlap between AAo_max_ and AAo_min_ as indicated by the strong observational and genetic correlations, as well as the large number of shared loci. The SNP-based heritability estimates for AAo_max_ and AAo_min_ were approximately 50%, whereas a previous twin study reported a heritability between 0·67 and 0·82 for the diameter of various parts of the aorta.^63^ This indicates a substantial proportion of missing heritability, analogous to previous studies of complex phenotypes.^64^ Missing heritability may be caused by the analysis of common variants in the GWAS and the inclusion of individuals who are primarily from European ancestry. Furthermore, due to the nature of GWAS, assuming an additive effects model, potential epistatic effects and gene-environment interactions may be overlooked.^64^ Finally, copy number variants could potentially play a role. However, considering genotype data used for the current GWAS only captures indels, but not copy number variants in general.^64^ Nonetheless, the heritability of 50% emphasizes the importance of common genetic variants in inter-individual differences in AAo anatomy. The *in-silico* annotations revealed biologically likely gene sets that were involved in cardiac and muscle structure development and pointed to the arteries as highest enriched tissue. The gene set enrichment analysis also showed the importance of cardiac development, with the top meta-clusters revolving around heart and muscle structure development. We found the strongest genetic overlap with blood pressure and anthropometric traits, although correlations were weak in general. We identified several genes that have been previously implicated to AAo anatomy or thoracic aneurysm development, including the *FBN1, ELN, MYH10, ULK4* and *SMG6* genes.^9^^,^^53^, ^54^, ^55^, ^56^, ^57^, ^58^, ^59^ We also replicated rs17470137, an intergenic variant annotated to *CCDC100* in a previous study.^10^ We annotated this variant to *PRDM6,* which encodes a histone methyltransferase and can alter epigenetic gene regulation.^65^^,^^66^ Alterations in *PRDM6* might influence AAo_max_ through regulation of vascular smooth muscle cells contractile proteins or through its association with patent ductus arteriosus.^65^^,^^66^

There was little overlap of the underlying biology between AAo function and anatomy as indicated by weak observational and genetic correlations, as well as the absence of overlap between the four SNPs associated with AAo function and those associated with AAo anatomy. The SNP-based heritability of AAo_dist_ was in line with previous GWASs, in which global arterial pressure measured by pulse wave velocity (PWV) was investigated, with estimates ranging between 0·06 and 0·28; all of which were lower than heritability estimated in twin studies (0·43–0·53).^67^, ^68^, ^69^, ^70^ *In-silico* annotations only highlighted blood serum as a significantly enriched tissue, possibly pointing to the role of circulating inflammatory markers in arterial stiffening.^71^^,^^72^ Again, we found the most significant genetic overlap with blood pressure and anthropometric traits. There was no overlap between the four identified loci in the current study and those highlighted in previous PWV GWASs.^69^^,^^70^^,^^73^ We note that the comparability with these previous studies might be limited considering they represent a different metric, as PWV reflects a global estimate rather than regional aortic stiffness. In fact, large ranges of SNP-based heritability and no replication of loci have been observed between PWV measurements.^69^^,^^70^

We searched for genetic variants which could alter protein encoding and identified a total of six genetic variants for the AAo traits was in high LD with coding variants of *JMJD1C, SMG6, NOC3L, ULK4*, and *ADAMTS7.* We evaluated their potential pathogenicity according to the ACMG guidelines and found that high relative CADD scores for coding variants of *ULK4* (rs3774372, in high LD with rs60248638) and *SMG6* (rs903160, in high LD with indel 17:2088848_CCAGA_C), providing one supportive argument for pathogenicity. In addition, we found that the prevalence of coronary artery disease was different between the alleles of 17:2088848_C_CCAGA, which provides evidence pathogenicity as well. However, the MR results did not support a causal relation between the investigated AAo traits and coronary artery disease development. Future research, such as functional studies and data on familial aggregation or de novo mutations in individuals with abnormal AAo traits, will be essential to provide definitive evidence on a potential causal role of these genes.

Multiple strategies were applied to search for candidate causal genes and we evaluated their molecular mechanisms of action to gain insights in their potential role in the biology underlying interindividual differences in AAo traits. We found several genes involved in connective tissue development (*COL6A3, ELN, FBN1*, and *THSD4),* which are of importance for the resistance of the aorta to stretching during the systolic phase*.* Both *FBN1,* which encodes fibrillin-1 and is associated with Marfan syndrome, and *ELN,* which encodes elastin and is associated with the cutis-laxa syndrome gene, are known for their importance in syndromic thoracic aneurysms.^53^, ^54^, ^55^, ^56^, ^57^ The *COL6A3* gene encodes the alpha-3 chain of type VI collagen and to date there are no studies which describe a role in AAo anatomy or function. Especially the role of type I and type III fibrillar collagens have been described for their role in the tensile strength of the aortic wall to withstand the high pressure of blood pumped by the heart, as they account for 80–90% of the total collagen present in the aorta.^74^^,^^75^ However, collagen type VI has also been found in intima and subintima of the human aorta and therefore play a role in aorta tensile strength.^76^ The *THSD4* gene encodes a microfibril-associated protein which binds to fibrillin-1. *In vitro* and *in vivo* experiments in mice have shown a role of *THSD4* in promoting fibrillin-1 its matrix assembly.^77^ We also identified multiple genes (*ADAMTS8, ANGPT1, ATF1, ECM1, FGD5, HAND2, HAS2, RNH1*) involved directly in angiogenesis. In addition, we highlighted genes involved in smooth muscle cell contraction (*ANO1, EDN1, EDN2, LPAR1*) and development (*ADAMTS7, PRDM6, MLK1*), which might play a role in arterial vasoconstriction. Of these genes, the *ADAMTS7* was identified by all gene-prioritization methods. The *ADAMTS7* gene encodes a zinc-dependent protease and has been associated with coronary artery disease in numerous GWAS.^78^^,^^79^ Another study showed an accumulation of *ADAMTS7* in smooth muscle cells in coronary and carotid atherosclerotic plaques.^80^ We also prioritized several genes involved in cardiac development, including *JMJD1C* which was identified by all gene-prioritization methods. *JMJD1C* encodes a histone demethylase, and mutations in this gene are associated with Rett Syndrome, a congenital neurological disorder.^81^ Rare deleterious single-nucleotide variations in *JMJD1C* have previously been linked to conotruncal type of congenital heart diseases commonly seen in individuals with 22q11·2 deletion or DiGeorge syndrome.^82^ Other potential biological mechanisms through which the identified genes could influence AAo anatomy and function included blood pressure regulation, atherogenesis and global cellular functions such as cell proliferation, differentiation, and migration.

Finally, we aimed to gain additional insights in the clinical consequences of AAo anatomy and function. The two-sample Mendelian randomization and sensitivity analyses provided robust evidence that genetically increased AAo_max_ and AAo_min_, as well as decreased AAo_dist_, increases risk of developing aneurysms. These results functionally validate that we captured AAo size biology. In addition, the high SNP-based heritability of AAo anatomy and its genetic association with aneurysm development emphasizes the role of genetics in aneurysm development.^5^^,^^83^ The results of the current study could be considered as further support for screening AAo size in a clinical setting, which previous studies have suggested for both familial and sporadic thoracic aneurysms.^8^^,^^84^ We did not find evidence for genetic associations with other cardiovascular diseases. This is in contrast to previous observational studies, which described that AAo_dist_ is associated with coronary artery disease and stroke.^12^^,^^85^ A biological explanation is that elastic arteries differ from muscular arteries in their relative ratios of smooth muscle cells, elastic fibres, and collagen.^86^ As reported before, smaller aorta diameters are correlated with stroke outcome. In a scenario where two individuals have equal compliance but different aortic size, the individual with the lower aortic size has a smaller reservoir volume and hence a higher pulse pressure. This could be the mechanism underlying the association between size and stroke since higher pulse pressure increases the risk of stroke.^87^^,^^88^ However, in the present study we did not find evidence for a causal relation between aortic size and stroke in the independent cohort. We did highlight some biological clusters that have been implicated in vascular disease pathophysiology, but found only little overlap with previously established loci and the correlations with coronary artery disease and stroke were weak. A statistical explanation for the absence of a genetic association between AAo_dist_ and cardiovascular disease outcomes is that the analyses might have been hampered by the inclusion of a limited number of genetic variants. To further our understanding of the relationship between aortic traits and cardiovascular outcomes, we, therefore, aim to continue our research on aortic traits, and to investigate the discrepancies between previous studies and the results in this study, with prospective follow-up on UK Biobank data, which consists of comprehensive health outcome data.

The key strength of the current study is that we used an AI-approach to analyse large quantities of CMR-data, which subsequently allowed us to perform a GWAS on AAo size and function in the largest cohort to date. Furthermore, we used an MR approach to overcome traditional sources of bias as confounding and reversed causation in traditional observational studies and performed a multitude of sensitivity analysis to prevent pleiotropic bias in the MR.^89^ Some limitations should be addressed as well.^90^ Firstly, we did not verify all images and predictions manually because of the large sample size and the large quantity of images. However, we carefully selected and verified the applied QC parameters by visual inspection of over 7500 images and corresponding segmentations, to prevent inclusion of incorrect images. Secondly, we were unable to differentiate between syndromic, familial and sporadic occurrences of AAo enlargement in the current analyses. We minimized the risk of false positives due to syndromic or familial cases through correction for familial structure in the GWAS and by excluding related individuals from the regression analyses that were performed to obtain the SNP-outcome associations that were used in the MR analyses.^28^ Thirdly, we were unable to replicate the newly discovered genetic variants associated with AAo anatomy and function in external cohorts as there are no equally sized cohorts with the combination of CMR and genetic data. We, therefore, hope to replicate these findings on an independent cohort in the future, by applying transfer learning on our AI based pipeline.

In conclusion, we developed an automated pipeline for segmentation of the AAo using an AI approach and used this to provide an overview of normal values of its dimensional and functional features in the largest population to date. We report 107 common genetic variants in 78 loci associated with AAo anatomy and function and found a high contribution of common genetic variants to AAo size. The Mendelian randomization analysis indicated a genetic association with aneurysm development, but not with other vascular diseases.

## Data sharing

The data that support the findings of this study are available from the corresponding author upon reasonable request. The GWAS datasets summary statistics generated during the current study are available in the following repository (https://doi.org/10.17632/dw7syw7tt7.1). Source data from UK biobank, CARDIoGRAMplusC4D and MEGASTROKE may be requested from the concerned consortia.

## Sources of funding

The work of J.W. Benjamins and M.W. Yeung was supported by the Research Project CVON-AI (2018B017), financed by the PPP Allowance made available by Top Sector Life Sciences & Health to the Dutch Heart Foundation to stimulate public-private partnerships. The work of N.Verweij was supported by NWO VENI grant 016.186.125.

## Contributors

All authors contributed to the study conception and design.

Formal analyses were performed by J.W.B., M.W.Y, Y.J.V. and M.A.S.

J.W.B., M.W.Y, Y.J.V., M.A.S., D.T., T.L., N.V. and P.H. were involved in interpreting the data.

Writing of the first draft was performed by J.W.B., M.W.Y, Y.J.V. and M.A.S.

Supervision, funding, manuscript reviewing, and editing were performed by L.J.O., N.V. and P.H.

All authors read and approved the final manuscript.

All authors take full responsibility for all aspects of the reliability and freedom from bias of the data presented and their discussed interpretation.

## Declaration of interests

Niek Verweij was employed by Regeneron Pharmaceuticals. Jan Walter Benjamins, Ming Wai Yeung, Yordi J. van de Vegte, M. Abdullah Said, Thijs van der Linden, Daan Ties, Luis E. Juarez-Orozco and Pim van der Harst declare that they have no competing interests.

## Supplementary materials

Image, application 1

Image, application 2

Image, application 3

Image, application 4
