# Supplementary material for: Genomic insights in ascending aortic size and distensibility
Source: eBioMedicine. 2021 Dec 28;75:103783. doi: 10.1016/j.ebiom.2021.103783 (PMC8718733; doi:10.1016/j.ebiom.2021.103783)
Supplement: Supplementary file 1 [file mmc1.docx]

**CLAIM: Checklist for Artificial Intelligence in Medical Imaging**

| Section / Topic | No. | Item |  |
| --- | --- | --- | --- |
| TITLE / ABSTRACT |  |  |  |
|  | **1** | Identification as a study of AI methodology, specifying the category of technology used (e.g., deep learning): **deep learning** | **✓** |
|  | **2** | Structured summary of study design, methods, results, and conclusions | **✓** |
| INTRODUCTION |  |  |  |
|  | **3** | Scientific and clinical background, including the intended use and clinical role of the AI approach | **✓** |
|  | **4** | Study objectives and hypotheses | **✓** |
| METHODS |  |  |  |
| *Study Design* | **5** | **Prospective** or retrospective study | **✓** |
|  | **6** | Study goal, such as model creation, exploratory study, feasibility study, non-inferiority trial | **✓** |
| *Data* | **7** | Data sources | **✓** |
|  | **8** | Eligibility criteria: how, where, and when potentially eligible participants or studies were identified (e.g., symptoms, results from previous tests, inclusion in registry, patient-care setting, location, dates) | **✓** |
|  | **9** | Data pre-processing steps | **✓** |
|  | **10** | Selection of data subsets, if applicable | **✓** |
|  | **11** | Definitions of data elements, with references to Common Data Elements | **✓** |
|  | **12** | De-identification methods | **NA** |
|  | **13** | How missing data were handled | **✓** |
| *Ground Truth* | **14** | Definition of ground truth reference standard, in sufficient detail to allow replication | **✓** |
|  | **15** | Rationale for choosing the reference standard (if alternatives exist) | **✓** |
|  | **16** | Source of ground-truth annotations; qualifications and preparation of annotators | **✓** |
|  | **17** | Annotation tools | **✓** |
|  | **18** | Measurement of inter- and intrarater variability; methods to mitigate variability and/or resolve discrepancies | **✓** |
| *Data Partitions* | **19** | Intended sample size and how it was determined | **✓** |
|  | **20** | How data were assigned to partitions; specify proportions | **✓** |
|  | **21** | Level at which partitions are disjoint (e.g., **image**, study, patient, institution) | **✓** |
| *Model* | **22** | Detailed description of model, including inputs, outputs, all intermediate layers and connections | **✓** |
|  | **23** | Software libraries, frameworks, and packages | **✓** |
|  | **24** | Initialization of model parameters (e.g., randomization, transfer learning) | **✓** |
| *Training* | **25** | Details of training approach, including data augmentation, hyperparameters, number of models trained | **✓** |
|  | **26** | Method of selecting the final model | **✓** |
|  | **27** | Ensembling techniques, if applicable | **NA** |
| *Evaluation* | **28** | Metrics of model performance | **✓** |
|  | **29** | Statistical measures of significance and uncertainty (e.g., confidence intervals) | **✓** |
|  | **30** | Robustness or sensitivity analysis | **✓** |
|  | **31** | Methods for explainability or interpretability (e.g., saliency maps), and how they were validated  **Image segmentation, we have thoroughly performed both validation by determining dice metric, but also visual verifications were performed by multiple human observers** | **NA** |
|  | **32** | Validation or testing on external data | **✓** |
| RESULTS |  |  |  |
| *Data* | **33** | Flow of participants or cases, using a diagram to indicate inclusion and exclusion | **✓** |
|  | **34** | Demographic and clinical characteristics of cases in each partition | **✓** |
| *Model performance* | **35** | Performance metrics for optimal model(s) on all data partitions | **✓** |
|  | **36** | Estimates of diagnostic accuracy and their precision (such as 95% confidence intervals) | **✓** |
|  | **37** | Failure analysis of incorrectly classified cases | **✓** |
| DISCUSSION |  |  |  |
|  | **38** | Study limitations, including potential bias, statistical uncertainty, and generalizability | **✓** |
|  | **39** | Implications for practice, including the intended use and/or clinical role | **✓** |
| OTHER INFORMATION |  |  |  |
|  | **40** | Registration number and name of registry | **✓** |
|  | **41** | Where the full study protocol can be accessed | **✓** |
|  | **42** | Sources of funding and other support; role of funders | **✓** |

Mongan J, Moy L, Kahn CE Jr. Checklist for Artificial Intelligence in Medical Imaging (CLAIM): a guide for authors and reviewers. Radiol Artif Intell 2020; 2(2):e200029. <https://doi.org/10.1148/ryai.2020200029>
