# Supplementary material for: Genomic insights in ascending aortic size and distensibility
Source: eBioMedicine. 2021 Dec 28;75:103783. doi: 10.1016/j.ebiom.2021.103783 (PMC8718733; doi:10.1016/j.ebiom.2021.103783)
Supplement: Supplementary file 3 [file mmc3.docx]

## Genomic insights in Ascending Aortic Size and Distensibility

Jan Walter Benjamins^1^, Ming Wai Yeung^1^, Yordi J. van de Vegte^1^, M. Abdullah Said^1^, Thijs van der Linden^1^, Daan Ties^1^, Luis E. Juarez-Orozco^1,2^, Niek Verweij^1^, Pim van der Harst^1,2^

1. University of Groningen, University Medical Center Groningen, Department of Cardiology, Groningen, The Netherlands

2. University Medical Center Utrecht, Department of Heart and Lungs, University of Utrecht, Utrecht, The Netherlands

## Index

**Supplementary methods**

**Supplementary Figures**

**Supplementary Figure 1:** Example of a cardiac cine image in a transverse cut at the level of the pulmonary trunk and the right pulmonary artery, as it was downloaded from UK biobank and used in this study, with on the right the annotation as it was predicted by the U-Net model.

**Supplementary Figure 2:** Split violin plot showing the distributions of AAo size and function for males and females, stratified by age.

**Supplementary Figure 3:** Quantile–quantile (QQ) plot for AAo_max_, AAo_min_ and AAo_dist_.

**Supplementary Figure 4:** Bar chart of the tissue enrichment analyses by DEPICT.

**Supplementary Figure 5:** Venn diagram showing the overlap of identified genes for AAo_max_, AAo_min_ and AAo_dist_.

**Supplementary Figure 6:** Venn diagram showing the overlap of genes identified in the current article and those that have previously been implicated in thoracic aorta size or thoracic aneurysm development.

**Supplementary Tables**

**Supplementary Table 1:** Overview of genes previously been implicated in thoracic aorta size or thoracic aneurysm development.

**Supplementary References**

**Supplementary Data**

**Supplementary Data 1:** Summary statistics of the 107 Genome-wide significant SNPs associated with AAo size and function.

**Supplementary Data 2:** List of AAo size and function variants associated with previously discovered variants.

**Supplementary Data 3:** Genetic correlation between AAo size and function and previously performed GWAS’s.

**Supplementary Data 4:** List of coding variants.

**Supplementary Data 5:** List of DEPICT genes.

**Supplementary Data 6:** Annotation of 101 identified genes.

**Supplementary Data 7:** Results of gene set analysis by DEPICT for AAo size and function.

**Supplementary Data 8:** Results of tissue enrichment analysis by DEPICT for AAo size and function.

**Supplementary Data 9:** Results of the Mendelian randomization analysis between AAo size and function and vascular diseases.

**Supplementary Data 10:** Additional sensitivity analyses of the Two-sample Mendelian randomization analyses of AAo size and function and cardiovascular diseases.

**Supplementary Data 11:** Phenotype definitions of mortality and cardiovascular disease phenotypes in the UK Biobank.

## Supplementary Data 12: Definitions of cardiovascular disease phenotypes in the CARDIoGRAMplusC4D and MEGASTROKE consortia.

## Supplementary Data 13: Differences in prevalence of lead SNPs in LD with coding variants in order to estimate potential pathogenicity

## Results

### Genome-wide analyses of ascending aorta dimensions

GWAS’s of AAo anatomy and function were performed in up to 35,110 individuals using 19,400,418 directly genotyped and imputed autosomal genetic variants. The GWAS revealed a total of 107 variants in 78 loci for all three AAo traits (***Online Table 1***). We found 65 SNPs in 59 loci for AAo_max_ area, 71 SNPs in 64 loci for AAo_min_ and 4 SNPs in 4 loci for AAo_dist_ ***(Figure 2****)****.*** In line with the high observational correlation between the phenotypes, out of 76 total loci, we found 47 shared loci between AAo_max_ and AAo_min_. In addition, AAo_max_ and AAo_min_ showed an almost perfect genetic correlations (r_g_ = 0·994 ± 0·002), while their respective correlation with AAo_dist_ was lower (r_g_ = -0·307 ± 0·054; r_g_ = -0·381 ± 0·047). There was little evidence of genomic inflation with genomic inflation lambdas of 1·20 for AAo_max_, 1·20 for AAo_min_, and 1·05 for AAo_dist_. This was further supported by the LD score regression intercepts of AAo_max_ (1·030 ± 0·0010), AAo_min_ (1·042 ± 0·010) and AAo_dist_ (1·022 ± 0·007). QQ-plots for AAo traits can be found in ***Supplementary Figure 3***. BOLT-REML estimated high SNP-based heritability for both AAo_max_ (h^2^_g_ = 0·509 ± 0·019) and AAo_min_ (h^2^_g_ = 0·502 ± 0·019), but a lower heritability for AAo_dist_ (h^2^_g_ = 0·151 ± 0·020). The identified variants explained 10·2 percent variance of AAo_max_, 10·7 percent for AAo_min_ and 0·7 percent for AAo_dist_. One standard deviation (SD) increase of the genetic risk score of AAo_max_, AAo_min_ and AAo_dist_ translated to an increase of 0·31 cm^2^, 0·31 cm^2^ and 0·07 × 10^-3^ mmHg^-1^ of the corresponding phenotype, respectively.

### Embedment in previous genetic studies

For each trait analyzed in the current GWAS, the GWAS catalog was used to search for previously identified SNPs in linkage disequilibrium with the SNPs discovered in the current study. Of the total of 107 SNPs discovered in the current study, we found 31 SNPs in high LD (*R*^2^ > 0. 8) with any previously assessed trait at a *P*-value threshold of *P* < 1 × 10^−5^ (***Online Table 2***). We found that the SNPs identified in the current study were in high LD (*R*^2^ > 0·8) with previously established variants for aortic size or thoracic aneurysms (four SNPs), blood pressure traits (12 SNPs) and atherosclerosis and thrombosis (two SNPs). LxDhub was queried to assess genetic correlations with other traits and showed that AAo traits were mainly correlated with blood pressure and anthropometric traits (***Online Table 3***).

### Candidate causal genes

We identified a total of 101 candidate causal genes for all 107 genetic variants (***Online Table 1***). A total of 86 unique genes were identified by proximity to the lead variant, defined as the nearest gene as well as any additional gene within 10 kb (***Online Table 1***). We identified five unique coding variants in high linkage disequilibrium with AAo size and function (***Online Table 1*** and ***Online Table 4***). DEPICT prioritized 44 unique genes while taking into account gene-gene similarities across loci (***Online Table 1*** and ***Online Table 5***). All three methods prioritized *JMJD1C* and *ADAMTS7* (***Figure*** ***4***)*.* Genes identified for AAo_max_ (72 genes) and AAo_min_ (81 genes) showed a large amount of overlap with 53 shared genes. We identified four genes for AAo_dist_. One gene, *ELN,* was shared between all AAo traits (***Supplementary Figure*** *5*). Annotation and further information of all identified genes is provided in ***Online Table 6***. We compared the identified genes with those that have previously been associated with syndromic, familial and sporadic aneurysms (***Supplementary Table 1, Online Figure 5***), and *FBN1, ELN*, *MYH10* and *ULK4* showed overlap with these previously implicated genes ^1–7^.

### Pathway and tissue enrichment analysis

Pathway enrichment analysis revealed 223 reconstituted gene sets in 30 gene clusters for AAo_max_, 419 significantly associated reconstituted gene sets located in 53 gene clusters for AAo_min_, but not a single reconstituted gene set for AAo distenstibility. A total of 178 reconstituted gene sets were significantly enriched for both AAo_max_ and AAo_min_. For AAo_max_, the central nodes of the top gene clusters were complete embryonic lethality during organogenesis, response to nutrient levels and circulatory system processes (***Online Table 7***). The central nodes of the top gene clusters for AAo_min_ were the CREBBP PPI subnetwork, smooth muscle cell proliferation and anemia. Tissue enrichment analysis revealed six significantly associated tissues for AAo_max_ and 10 significantly associated tissues for AAo_min_. We found no significantly enriched tissues for AAo_dist_. Both AAo_max_ and AAo_min_ were strongest enriched in the arteries and the top five tissues mainly revolved around the cardiovascular system for both AAo_max_ and AAo_min_. Full results of the tissue enrichment analysis can be found in ***Online Table 8*** and are shown in ***Supplementary Figure 4***.

### Mendelian randomization analysis

A series of Two-sample MR analyses was performed to investigate the potential causal mechanisms between AAo size and function and cardiovascular diseases. We focused on cardiovascular diseases in which alterations in arterial vessel wall biology might contribute to disease pathophysiology, including CAD, myocardial infarction, stroke, and aneurysms. We assumed balanced horizontal pleiotropy in the MR analyses and therefore adopted the inverse variance weighted random (IVW-RE) effects model as most liberal. Please see ***Online Table 9*** for the full MR results and ***Online Table 10*** for the sensitivity analyses. Disease definitions are detailed in ***Online Table 11*** for the UK Biobank and in ***Online Table 12*** for the CARDIoGRAMplusC4D and MEGASTROKE consortia. The IVW-RE, MR-Lasso and weighted median estimates for all cardiovascular diseases tested within the UK Biobank are shown in ***Figure 3 (left panel)*** and those assessed in the CARDIoGRAMplusC4D and MEGASTROKE consortia in ***Figure 3 (right panel)****.*

First, we assessed the association between AAo size and function and ischemic heart diseases. We found no evidence for an association between AAo_max_, AAo_min_ or AAo_dist_ and coronary artery disease or myocardial infarction in the UK Biobank or in the CARDIoGRAMplusC4D cohort using an inverse variance weighted random-effects model (***Figure 3****)*.

We then sought to determine possible causal relationships between AAo anatomy and distensibility and stroke. We found that AAo_max_ and AAo_min_ were suggestively associated with ischemic stroke (OR 1·109, CI 1·031 − 1·192, *P* = 5·30 × 10^-3^; OR 1·107, CI 1·032 − 1·188, *P* = 4·35 × 10^-3^, respectively). We aimed to replicate this finding in the MEGASTROKE consortium and found no evidence that increased AAo_max_ and AAo_min_ increase risk of any stroke (OR 1·004, CI 0·949 − 1·062, *P* = 8·93 × 10^-1^; OR 1·042, CI 0·992 − 1·095, *P* =1·02 × 10^-1^, respectively). We also found suggestively significant associations between AAo_min_ any stroke in the UK Biobank, but again the results were not replicated in the MEGASTROKE consortium (***Figure 3***). AAo_dist_ was not associated with any or ischemic stroke (***Figure 3***). We did not find evidence for an association between AAo anatomy and distensibility and other stroke subtypes, including subarachnoidal haemorrhage, intracerebral haemorrhage, cardio-embolic, large-artery and small-vessel stroke (***Online Table 10***).

Lastly, we studied the association between AAo size and function and aneurysms. We found strong evidence for a causal association between AAo_max_, AAo_min_, AAo_dist_ and aneurysms (OR 1·557, CI 1·328 − 1·825, *P* = 4·67 × 10^-8^; OR 1·500, CI 1·287 – 1·748, *P* = 2·24 × 10^-7^; 0·453, CI 0·243 − 0·780, *P =* 4·70 × 10^-3^, respectively). The causal estimates were robust to MR-Lasso and weighted median analyses (***Figure 3***), two complementary sensitivity analyses which provides a true causal estimate under the scenarios that respectively several or up to half of the variants are invalid.

We lowered the *P-*value threshold for inclusion of genetic variants to *P* < 1 × 10^-6^, considering the low number of genetic variants discovered for AAo_dist_. The results were generally consistent with the main results for all exposure-outcome associations (***Online Table 10***).

**Supplementary Figure 1:** Example of a cardiac cine image in a transverse cut at the level of the pulmonary trunk and the right pulmonary artery, as it was downloaded from UK biobank and used in this study, with on the right the annotation as it was predicted by the U-Net model.

**
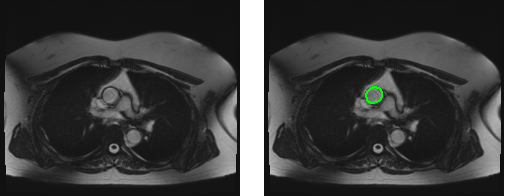
**

**Supplementary Figure 2:** Split violin plot showing the distributions of AAo size and function for males and females, stratified by age.


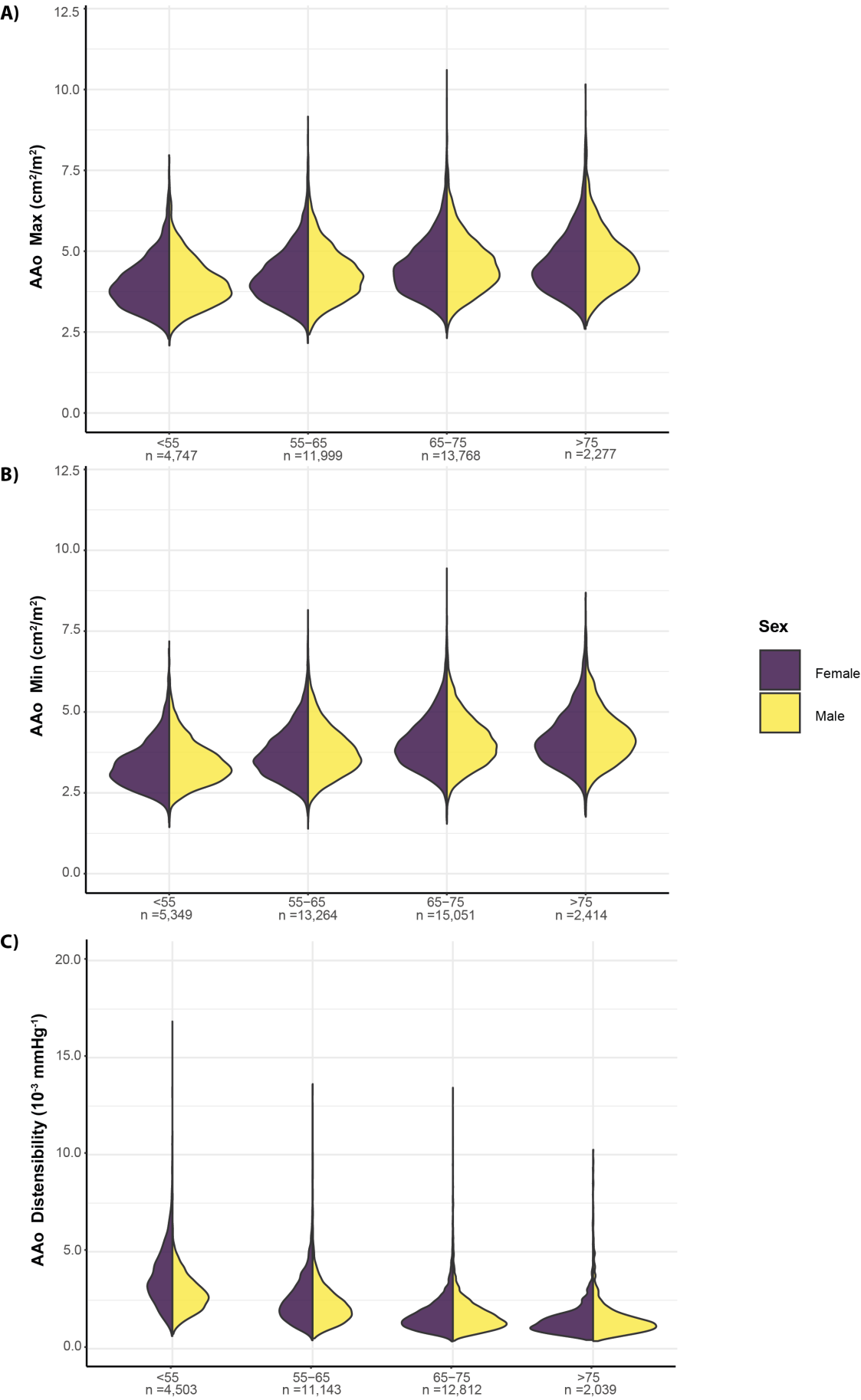


*Split violin plots of A) AAo_max_, B) AAo_min_ and C) AAo_dist_* *for males and females, stratified by age. The X-axis shows the age-category and the amount of individuals represented in the age-category. The Y-axis show A) AAo_max_ in cm^2^/m^2^, B) AAo_min_ in cm^2^/m^2^ and C) AAo_dist_ in 10^-3^ mmHg^-1^.The distribution of AAo size and function is shown in purple for women and in yellow for men.*

**Supplementary Figure 3:** Quantile–quantile (QQ) plot for AAo_max_, AAo_min_ and AAo_dist_.


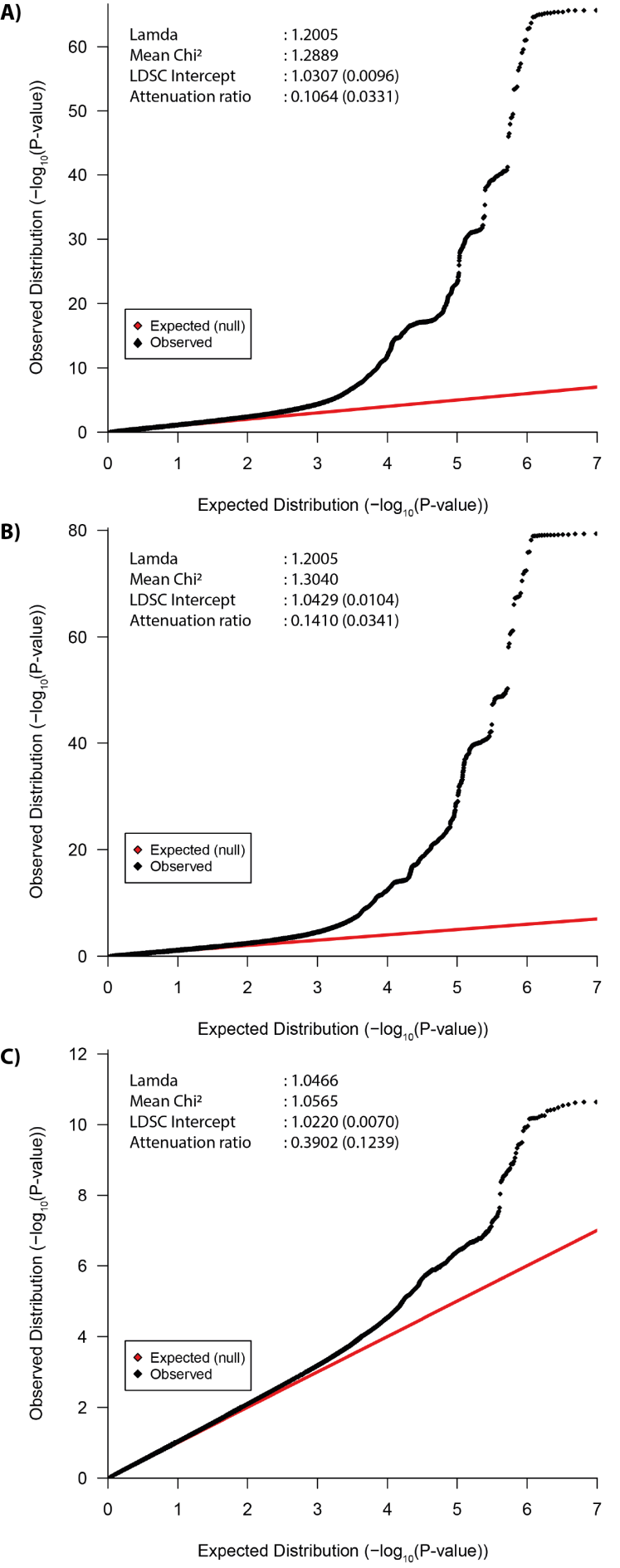


*QQ-plots of the GWAS’s of A) AAo_max_, B) AAo_min_ and C) AAo_dist_. The X-axis shows the expected distribution in –log_10_(P-value). The Y-axis the observed distribution in –log_10_(P-value). The red line follows expected P-values from a theoretical χ2 -distribution, whereas the black line follows the observed P-values in the current GWAS’s.*

**Supplementary Figure 4:** Bar chart of the tissue enrichment analyses by DEPICT.

*
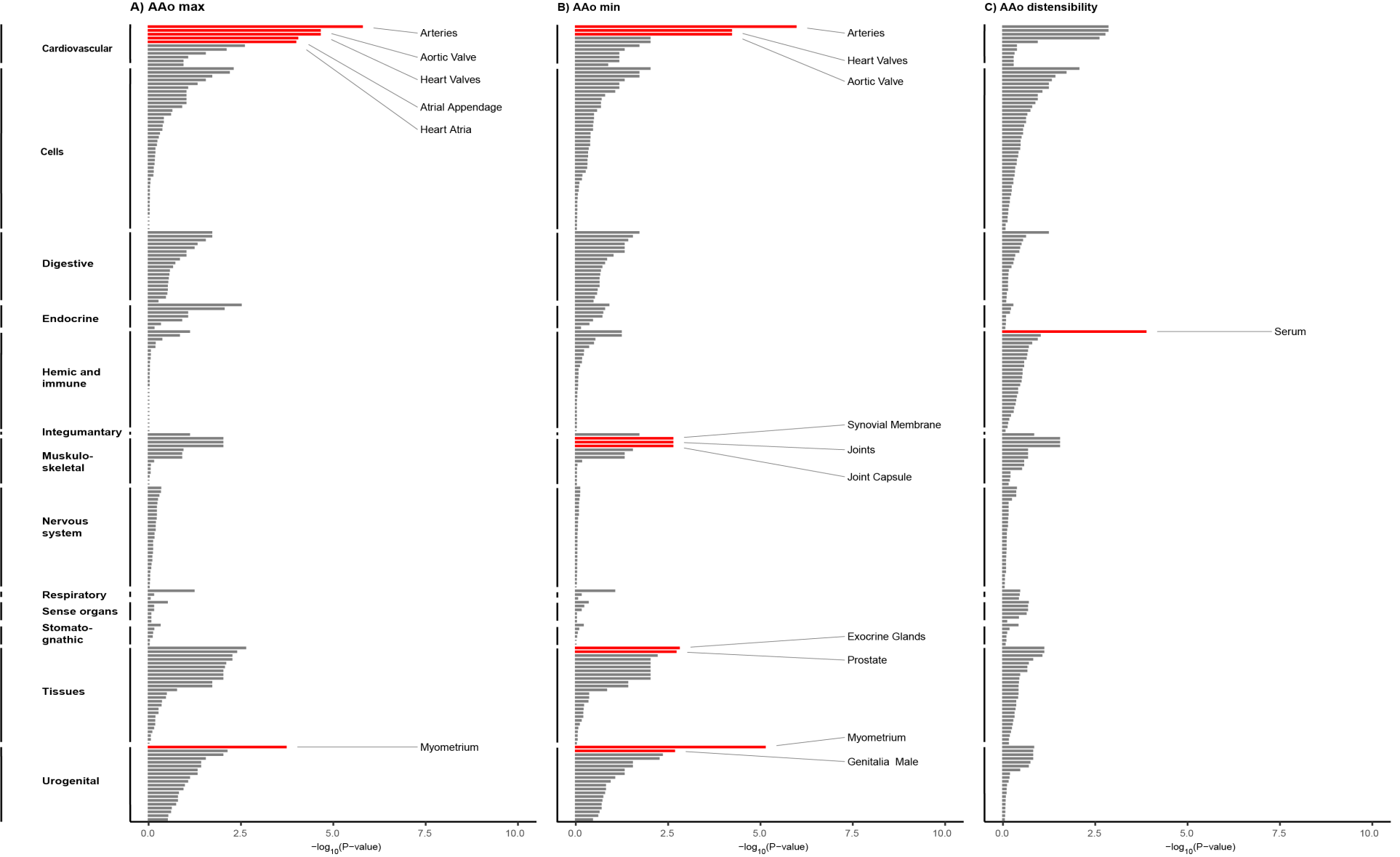
*

*Results of the DEPICT tissue enrichment analysis for A) AAo_max_, B) AAo_min_ and C) AAo_dist_. The Y-axis shows the tissues clustered by first MeSH term, ordered on the –log_10_(P-value) per cluster. The X-axis shows the –log_10_(P-value). An FDR <0.05 was considered to be statistically significant and are . plotted in red and annotated, other tissues are plotted in grey.*

**Supplementary Figure 5:** Venn diagram showing the overlap of identified genes for AAo_max_, AAo_min_ and AAo_dist_.


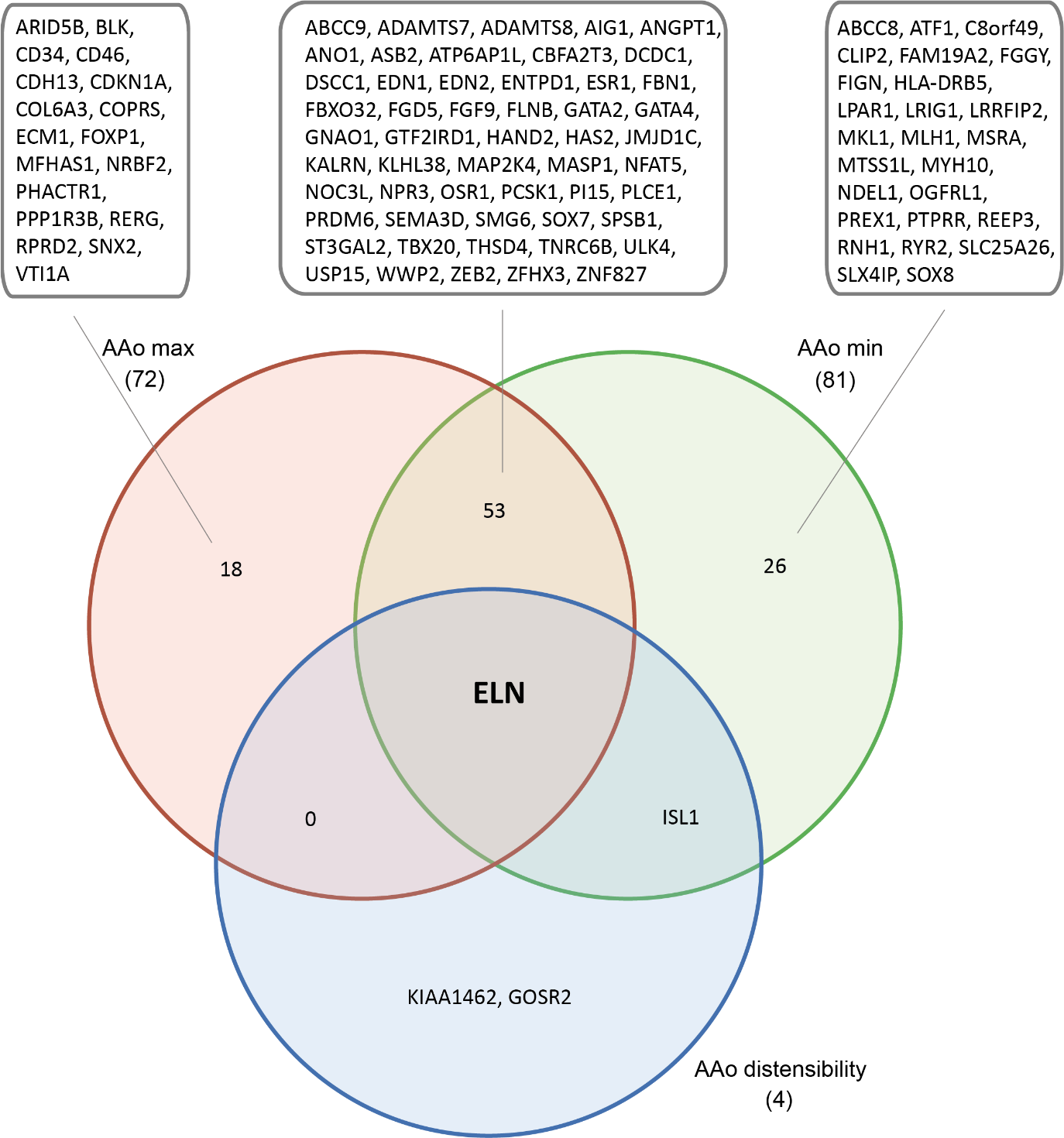


*The Venn plot shows overlap between loci found for AAo_max_, AAo_min_ and AAo_dist_. Many genes are shared between AAo_max_ and AAo_min_, which also showed high observational and genetic correlations. The ELN gene was shared between all traits.*

**Supplementary Figure 6:** Venn diagram showing the overlap of identified genes for AAo_max_, AAo_min_ and AAo_dist_.


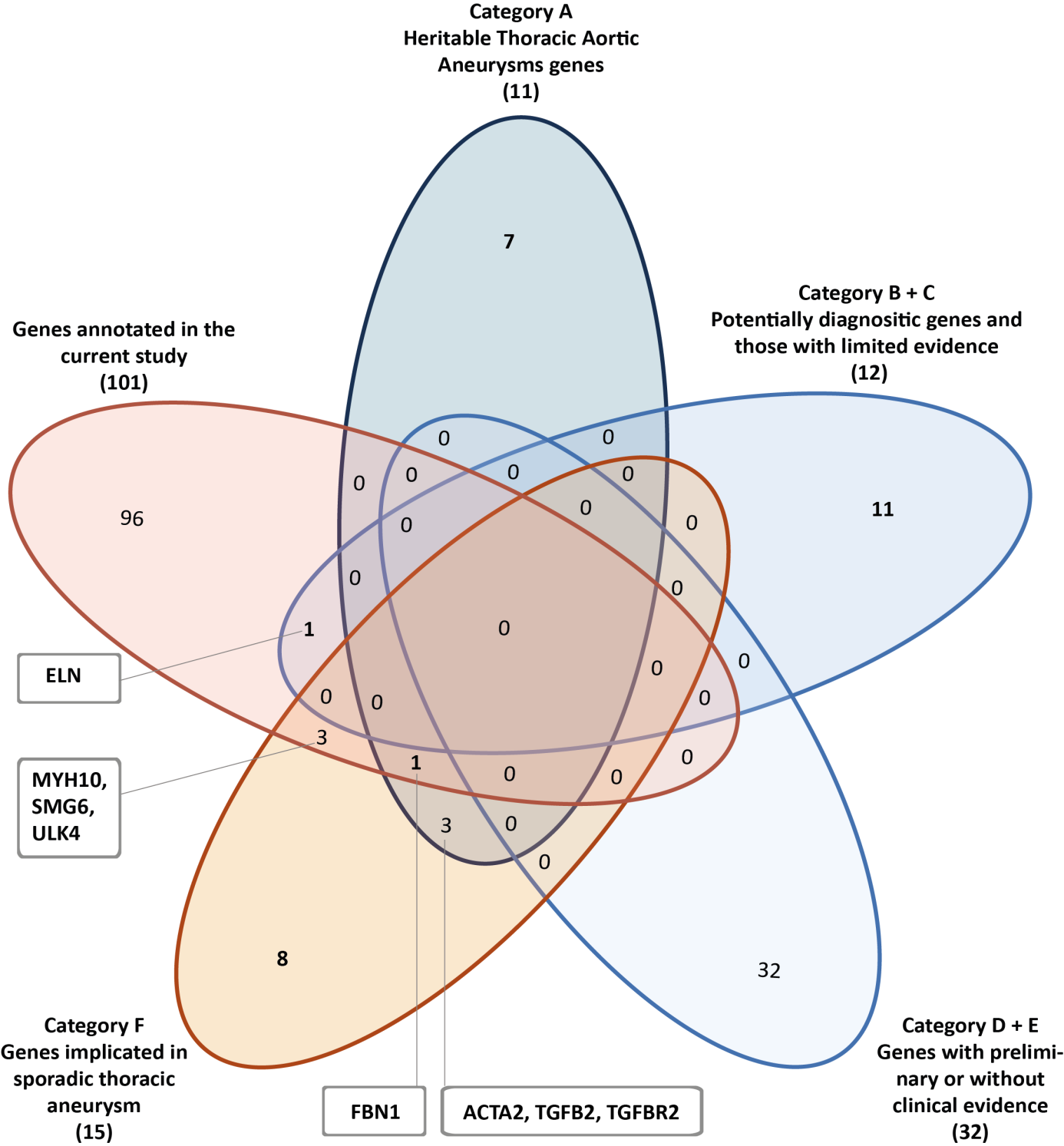


*The Venn plot shows overlap between loci found in the current GWAS’s of AAo size and function (AAo_max_, AAo_min_ and AAo_dist_) and genes that have previously been implicated syndromic, familial and sporadic heritable thoracic aortic aneurysms in humans, based on the ClinGen Aortopathy Working Group classification*^8^**.** *Categories A comprises genes designated as heritable thoracic aortic aneurysms genes. In A1 syndromic entities co-occur. Category B are genes labeled as “Potentially diagnostic genes”. These may allow diagnosis of the cause of thoracic aortic enlargement, but are primarily associated with other clinical features and which do not carry relevant risks of progression to aortic dissection. Category C comprises genes with limited evidence in which diagnosis is primarily based on nonvascular features. Category D comprises genes for which some experimental data may suggest a link with thoracic aortic disease, but no clinical evidence is available. Category E comprises genes for which the data are recent and preliminary, and no accurate categorization is possible at present. We added category F, which comprises genes that have previously been associated with sporadic thoracic aortic aneurysms or AAo size.*

**Supplementary Table 1:** Overview of genes previously been implicated in thoracic aorta size or thoracic aneurysm development.

| **Gene** | **Phenotype (omim number)** | **Reference** |
| --- | --- | --- |
| **Category A1: Heritable Thoracic Aortic Aneurysms and Dissections, syndromic** | | |
| *COL3A1* | Ehlers-Danlos syndrome, vascular type (120180) | ^9^ |
| *FBN1* | Marfan syndrome (134797) | ^5^ |
| *SMAD3* | Loeys-Dietz syndrome 3 (603109) | ^10^ |
| *TGFB2* | Loeys-Dietz syndrome 4 (190220) | ^11^ |
| *TGFBR1* | Loeys-Dietz syndrome 1 (190181) | ^12^ |
| *TGFBR2* | Loeys-Dietz syndrome 2 (190182) | ^12^ |
| **Category A2: Heritable Thoracic Aortic Aneurysms and Dissections, non-syndromic** | | |
| *ACTA2* | Multisystemic smooth muscle dysfunction syndrome; Aortic aneurysm, familial thoracic 6 (102620) | ^13,14^ |
| *MYH11* | Aortic aneurysm, familial thoracic 4 (160745) | ^15,16^ |
| *MYLK* | Aortic aneurysm, familial thoracic 7 (600922) | ^17^ |
| *LOX* | Aortic aneurysm, familial thoracic 10 (153455) | ^18^ |
| *PRKG1* | Aortic aneurysm, familial thoracic 8 (176894) | ^19^ |
| **Category B : Potentially diagnostic genes** | | |
| *EFEMP2* | Cutis laxa, autosomal recessive, type IB (604633) | ^20,21^ |
| *ELN* | Cutis Laxa, Autosomal Dominant (130160) | ^1–4^ |
| *FBN2* | Contractural Arachnodactyly, Congenital (612570) | ^22–26^ |
| *FLNA* | Periventricular Nodular Heterotopia (300017) | ^27–30^ |
| *NOTCH1* | Aortic valve disease 1 (190198) | ^31–38^ |
| *SLC2A10* | Arterial Tortuosity syndrome (606145) | ^1,39–42^ |
| *SMAD4* | Juvenile polyposis syndrome (600993) | ^43–46^ |
| *SKI* | Sphrintzen-Goldberg Craniosynostosis syndrome (164780) | ^47,48^ |
| **Category C : Limited evidence** | | |
| *CBS* | Homocystinuria (236200) | ^49^ |
| *COL4A5* | Alport syndrome, X-linked (303630) | ^50^ |
| *PKD1* | Polycistic kidney disease, type 1 (601313) | ^51^ |
| *PKD2* | Polycistic kidney disease, type 2 (173910) | ^51^ |
| **Category D: No evidence** | | |
| *ACVRL1* | Telangiectasia, hereditary hemorrhagic, type 2 (601284) | - |
| *ADAMTS10* | Weill-Marchesani syndrome 1, recessive (608990) | - |
| *B3GAT3* | Multiple joint dislocations, short stature, craniofacial dysmorphism, with or without congenital heart defects (606374) | - |
| *COL1A1* | Osteogenesis Imperfecta, type I (120150) | - |
| *COL1A2* | Ehlers-Danlos, cardiac valvular form (120160) | - |
| *COL4A1* | Angiopathy, hereditary, with nephropathy, aneurysms and muscle cramps (120130) | - |
| *COL5A1* | Ehlers-Danlos, type I (120215) | - |
| *COL5A2* | Ehlers-Danlos, type I (120190) | - |
| *COL9A1* | Stickler syndrome, type IV (120210) | - |
| *COL9A2* | Stickler syndrome, type V (120260) | - |
| *COL11A1* | Stickler syndrome, type II (120280) | - |
| *COL18A1* | Knobloch syndrome, type I (120328) | - |
| *EMILIN1* | Extracellular matrix glycoprotein localized at sites where elastin and microfibrils are in proximity. (130660) | - |
| *ENG* | Telangiectasia, hereditary hemorrhagic, type 1 (131195) | - |
| *GATA5* | Congenital heart defects, multiple types, 5 (611496) | - |
| *GJA1* | Atrioventricular septal defect 3 (121014) | - |
| *JAG1* | Alagille syndrome (601920) | - |
| *MED12* | Lujan-Fryns syndrome (300188) | - |
| *PLOD1* | Ehlers-Danlos syndrome, type VI (153454) | - |
| *PLOD3* | Lysyl hydroxylase 3 defiency (603066) | - |
| *SMAD6* | Aortic valve disease 2 (602931) | - |
| *UPF3B* | Mental retardation, X-linked, syndromic 14 (300298) | - |
| *VCAN* | Wagner syndrome 1 (118661) | - |
| **Category E: Uncertain** |  |  |
| *BGN* | Meester-Loeys syndrome (301870) | ^52^ |
| *FOXE3* | Aortic aneurysm, familial thoracic 11, susceptibility to (601094) | ^53^ |
| *HCN4* | Sick sinus syndrome 2 (605206) | ^54^ |
| *MAT2A* | Methionine adenosyltransferase (601468) | ^55^ |
| *MFAP5* | Aortic aneurysm, familial thoracic 9 (601103) | ^56,57^ |
| *SMAD2* | Mothers against decapentaplegic, drosophila (601366) | ^58–60^ |
| *TGFB3* | Loeys-Dietz syndrome 5 (190230) | ^59,61^ |
| *ARIH1* | Ariadne drosophila homolog 1 (605624) | ^62^ |
| *LTBP3* | Dental anomalies and short stature syndrome (602090) | ^63^ |
| **Category F: Implicated in sporadic TAAD or AAo size** | | |
| *FBN1* | Marfan syndrome (134797) | ^64^ |
| *ULK4* | Serine/threonine kinases (617010) | ^6^ |
| *CLU* | Sulfated glycoprotein 2 (185430) | ^7^ |
| *DES* | Cardiomyopathy, dilated, 1I (125660) | ^7^ |
| *MYH10* | Myosin, heavy chain, nonmuscle; MYH10 (160776) | ^7^ |
| *FBLN5* | Cutis laxa, autosomal dominant 2; Cutis laxa, autosomal recessive, type IA (604580) | ^7^ |
| *COL3A* | Leprosy, paucibacillary type, susceptibility to (609888) | ^65^ |
| *SMAD* | Signal transducers for receptors of the transforming growth factor beta (TGF-B) superfamily (605532) | ^65^ |
| *ACTA2* | Aortic aneurysm, familial thoracic 6 (102620) | ^65^ |
| *TGFB2* | Loeys-Dietz syndrome 4 (190220) | ^65^ |
| *TGFBR2* | Loeys-Dietz syndrome 2 (190182) | ^65^ |
| *SMG6* | nonsense-mediated mRNA decay (610963) | ^66^ |
| *CCDC100* | Joubert syndrome (613446) | ^66^ |
| *HMGA2* | Silver-Russell syndrome 5 (618908) | ^66^ |
| *PDE3A* | Hypertension and brachydactyly syndrome (123805) | ^66^ |

*Overview of genes that have previously been implicated in syndromic, familial and sporadic heritable thoracic aortic aneurysms in humans, based on the ClinGen Aortopathy Working Group classification*^8^*. The categories proposed by the ClinGen Aortopathy Working Group reflect the amount of evidence for the gene-disease relationships*^8^*. Categories A comprises genes designated as heritable thoracic aortic aneurysms genes. In A1 syndromic entities co-occur. Category B are genes labeled as “Potentially diagnostic genes”. These may allow diagnosis of the cause of thoracic aortic enlargement, but are primarily associated with other clinical features and which do not carry relevant risks of progression to aortic dissection. Category C comprises genes with limited evidence in which diagnosis is primarily based on nonvascular features. Category D comprises genes for which some experimental data may suggest a link with thoracic aortic disease but no clinical evidence is available. Category E comprises genes for which the data are recent and preliminary and no accurate categorization is possible at present. We added category F, which comprises genes that have previously been associated with sporadic thoracic aortic aneurysms or AAo size.*

## Supplementary references

1 Callewaert B, Renard M, Hucthagowder V, *et al.* New insights into the pathogenesis of autosomal-dominant cutis laxa with report of five ELN mutations. *Hum Mutat* 2011; **32**: 445–55.

2 Vodo D, Sarig O, Peled A, Frydman M, Greenberger S, Sprecher E. Autosomal-dominant cutis laxa resulting from an intronic mutation in ELN. *Exp Dermatol* 2015; **24**: 885–7.

3 Hadj-Rabia S, Callewaert BL, Bourrat E, *et al.* Twenty patients including 7 probands with autosomal dominant cutis laxa confirm clinical and molecular homogeneity. *Orphanet J Rare Dis* 2013; **8**. DOI:10.1186/1750-1172-8-36.

4 Szabo Z, Crepeau MW, Mitchell AL, *et al.* Aortic aneurysmal disease and cutis laxa caused by defects in the elastin gene. *J Med Genet* 2006; **43**: 255–8.

5 Dietz HC, Cutting CR, Pyeritz RE, *et al.* Marfan syndrome caused by a recurrent de novo missense mutation in the fibrillin gene. *Nature* 1991; **352**: 337–9.

6 Guo D chuan, Grove MLL, Prakash SKK, *et al.* Genetic Variants in LRP1 and ULK4 Are Associated with Acute Aortic Dissections. *Am J Hum Genet* 2016; **99**: 762–9.

7 Xu H, Chen S, Zhang H, *et al.* Network-based analysis reveals novel gene signatures in the peripheral blood of patients with sporadic nonsyndromic thoracic aortic aneurysm. *J Cell Physiol* 2020; **235**: 2478–91.

8 Renard M, Francis C, Ghosh R, *et al.* Clinical Validity of Genes for Heritable Thoracic Aortic Aneurysm and Dissection. *J Am Coll Cardiol* 2018; **72**: 605–15.

9 Legrand A, Devriese M, Dupuis-Girod S, *et al.* Frequency of de novo variants and parental mosaicism in vascular Ehlers–Danlos syndrome. *Genet Med* 2019; **21**: 1568–75.

10 Van De Laar IMBH, Oldenburg RA, Pals G, *et al.* Mutations in SMAD3 cause a syndromic form of aortic aneurysms and dissections with early-onset osteoarthritis. Nat. Genet. 2011; **43**: 121–6.

11 Lindsay ME, Schepers D, Bolar NA, *et al.* Loss-of-function mutations in TGFB2 cause a syndromic presentation of thoracic aortic aneurysm. *Nat Genet* 2012; **44**: 922–7.

12 Loeys BL, Schwarze U, Holm T, *et al.* Aneurysm Syndromes Caused by Mutations in the TGF-β Receptor. *N Engl J Med* 2006; **355**: 788–98.

13 Guo DC, Papke CL, Tran-Fadulu V, *et al.* Mutations in Smooth Muscle Alpha-Actin (ACTA2) Cause Coronary Artery Disease, Stroke, and Moyamoya Disease, Along with Thoracic Aortic Disease. *Am J Hum Genet* 2009; **84**: 617–27.

14 Guo DC, Pannu H, Tran-Fadulu V, *et al.* Mutations in smooth muscle α-actin (ACTA2) lead to thoracic aortic aneurysms and dissections. *Nat Genet* 2007; **39**: 1488–93.

15 Harakalova M, Van Der Smagt J, De Kovel CGF, *et al.* Incomplete segregation of MYH11 variants with thoracic aortic aneurysms and dissections and patent ductus arteriosus. *Eur J Hum Genet* 2013; **21**: 487–93.

16 Pannu H, Tran-Fadulu V, Papke CL, *et al.* MYH11 mutations result in a distinct vascular pathology driven by insulin-like growth factor 1 and angiotensin II. *Hum Mol Genet* 2007; **16**: 2453–62.

17 Wang L, Guo DC, Cao J, *et al.* Mutations in myosin light chain kinase cause familial aortic dissections. *Am J Hum Genet* 2010; **87**: 701–7.

18 Leea VS, Halabi CM, Hoffman EP, *et al.* Loss of function mutation in LOX causes thoracic aortic aneurysm and dissection in humans. *Proc Natl Acad Sci U S A* 2016; **113**: 8759–64.

19 Guo DC, Regalado E, Casteel DE, *et al.* Recurrent gain-of-function mutation in PRKG1 causes thoracic aortic aneurysms and acute aortic dissections. *Am J Hum Genet* 2013; **93**: 398–404.

20 Huang J, Davis EC, Chapman SL, *et al.* Fibulin-4 deficiency results in ascending aortic aneurysms: A potential link between abnormal smooth muscle cell phenotype and aneurysm progression. *Circ Res* 2010; **106**: 583–92.

21 Hucthagowder V, Sausgruber N, Kim KH, Angle B, Marmorstein LY, Urban Z. Fibulin-4: A novel gene for an autosomal recessive cutis laxa syndrome. *Am J Hum Genet* 2006; **78**: 1075–80.

22 Takeda N, Morita H, Fujita D, *et al.* Congenital contractural arachnodactyly complicated with aortic dilatation and dissection: Case report and review of literature. *Am J Med Genet Part A* 2015; **167**: 2382–7.

23 Gupta PA, Putnam EA, Carmical SG, *et al.* Ten novel FBN2 mutations in congenital contractural arachnodactyly: Delineation of the molecular pathogenesis and clinical phenotype. *Hum Mutat* 2002; **19**: 39–48.

24 Callewaert BL, Loeys BL, Ficcadenti A, *et al.* Comprehensive clinical and molecular assessment of 32 probands with congenital contractural arachnodactyly: Report of 14 novel mutations and review of the literature. *Hum Mutat* 2009; **30**: 334–41.

25 Gupta PA, Wallis DD, Chin TO, *et al.* FBN2 mutation associated with manifestations of Marfan syndrome and congenital contractural arachnodactyly. J. Med. Genet. 2004; **41**. DOI:10.1136/jmg.2003.012880.

26 Park E-S, Putnam EA, Chitayat D, Child A, Milewicz DM. Clustering ofFBN2 mutations in patients with congenital contractural arachnodactyly indicates an important role of the domains encoded by exons 24 through 34 during human development. *Am J Med Genet* 1998; **78**: 350–5.

27 Ziganshin BA, Bailey AE, Coons C, *et al.* Routine genetic testing for thoracic aortic aneurysm and dissection in a clinical setting. *Ann Thorac Surg* 2015; **100**: 1604–11.

28 Lange M, Kasper B, Bohring A, *et al.* 47 patients with FLNA associated periventricular nodular heterotopia. *Orphanet J Rare Dis* 2015; **10**: 134.

29 Reinstein E, Frentz S, Morgan T, *et al.* Vascular and connective tissue anomalies associated with X-linked periventricular heterotopia due to mutations in Filamin A. *Eur J Hum Genet* 2013; **21**: 494–502.

30 Sheen VL, Jansen A, Chen MH, *et al.* Filamin A mutations cause periventricular heterotopia with Ehlers-Danlos syndrome. *Neurology* 2005; **64**: 254–62.

31 Koenig SN, LaHaye S, Feller JD, *et al.* Notch1 haploinsufficiency causes ascending aortic aneurysms in mice. *JCI Insight* 2017; **2**. DOI:10.1172/jci.insight.91353.

32 Mohamed SA, Aherrahrou Z, Liptau H, *et al.* Novel missense mutations (p.T596M and p.P1797H) in NOTCH1 in patients with bicuspid aortic valve. *Biochem Biophys Res Commun* 2006; **345**: 1460–5.

33 McKellar SH, Tester DJ, Yagubyan M, Majumdar R, Ackerman MJ, Sundt TM. Novel NOTCH1 mutations in patients with bicuspid aortic valve disease and thoracic aortic aneurysms. *J Thorac Cardiovasc Surg* 2007; **134**: 290–6.

34 Garg V, Muth AN, Ransom JF, *et al.* Mutations in NOTCH1 cause aortic valve disease. *Nature* 2005; **437**: 270–4.

35 Mcbride KL, Riley MF, Zender GA, *et al.* NOTCH1 mutations in individuals with left ventricular outflow tract malformations reduce ligand-induced signaling. *Hum Mol Genet* 2008; **17**: 2886–93.

36 Foffa I, Ait Alì L, Panesi P, *et al.* Sequencing of NOTCH1, GATA5, TGFBR1 and TGFBR2 genes in familial cases of bicuspid aortic valve. *BMC Med Genet* 2013; **14**: 44.

37 Theis JL, Hrstka SCL, Evans JM, *et al.* Compound heterozygous NOTCH1 mutations underlie impaired cardiogenesis in a patient with hypoplastic left heart syndrome. *Hum Genet* 2015; **134**: 1003–11.

38 Kerstjens-Frederikse WS, Van De Laar IMBH, Vos YJ, *et al.* Cardiovascular malformations caused by NOTCH1 mutations do not keep left: Data on 428 probands with left-sided CHD and their families. *Genet Med* 2016; **18**: 914–23.

39 Faiyaz-Ul-Haque M, Zaidi SHE, Al-Sanna N, *et al.* A novel missense and a recurrent mutation in SLC2A10 gene of patients affected with arterial tortuosity syndrome. *Atherosclerosis* 2009; **203**: 466–71.

40 Callewaert BL, Willaert A, Kerstjens-Frederikse WS, *et al.* Arterial tortuosity syndrome: Clinical and molecular findings in 12 newly identified families. *Hum Mutat* 2008; **29**: 150–8.

41 Ritelli M, Drera B, Vicchio M, *et al.* Arterial tortuosity syndrome in two Italian paediatric patients. *Orphanet J Rare Dis* 2009; **4**: 20.

42 Coucke PJ, Willaert A, Wessels MW, *et al.* Mutations in the facilitative glucose transporter GLUT10 alter angiogenesis and cause arterial tortuosity syndrome. *Nat Genet* 2006; **38**: 452–7.

43 Jelsig AM, Tørring PM, Kjeldsen AD, *et al.* JP–HHT phenotype in Danish patients with SMAD4 mutations. *Clin Genet* 2016; **90**: 55–62.

44 Andrabi S, Bekheirnia MR, Robbins-Furman P, Lewis RA, Prior TW, Potocki L. SMAD4 mutation segregating in a family with juvenile polyposis, aortopathy, and mitral valve dysfunction. *Am J Med Genet Part A* 2011; **155**: 1165–9.

45 Teekakirikul P, Milewicz DM, Miller DT, *et al.* Thoracic aortic disease in two patients with juvenile polyposis syndrome and SMAD4 mutations. *Am J Med Genet Part A* 2013; **161**: 185–91.

46 Heald B, Rigelsky C, Moran R, *et al.* Prevalence of thoracic aortopathy in patients with juvenile Polyposis Syndrome-Hereditary Hemorrhagic Telangiectasia due to SMAD4. *Am J Med Genet Part A* 2015; **167**: 1758–62.

47 Doyle AJ, Doyle JJ, Bessling SL, *et al.* Mutations in the TGF-β repressor SKI cause Shprintzen-Goldberg syndrome with aortic aneurysm. *Nat Genet* 2012; **44**: 1249–54.

48 Carmignac V, Thevenon J, Adès L, *et al.* In-frame mutations in exon 1 of SKI cause dominant shprintzen-goldberg syndrome. *Am J Hum Genet* 2012; **91**: 950–7.

49 Lorenzini M, Guha N, Davison JE, *et al.* Isolated aortic root dilation in homocystinuria. *J Inherit Metab Dis* 2018; **41**: 109–15.

50 Kashtan CE, Segal Y, Flinter F, Makanjuola D, Gan J Sen, Watnick T. Aortic abnormalities in males with Alport syndrome. *Nephrol Dial Transplant* 2010; **25**: 3554–60.

51 Peczkowska M, Januszewicz A, Grzeszczak W, *et al.* The coexistence of acute aortic dissection with autosomal dominant polycystic kidney disease - Description of two hypertensive patients. *Blood Press* 2004; **13**: 283–6.

52 Meester JAN, Vandeweyer G, Pintelon I, *et al.* Loss-of-function mutations in the X-linked biglycan gene cause a severe syndromic form of thoracic aortic aneurysms and dissections. *Genet Med* 2017; **19**: 386–95.

53 Kuang SQ, Medina-Martinez O, Guo DC, *et al.* FOXE3 mutations predispose to thoracic aortic aneurysms and dissections. *J Clin Invest* 2016; **126**: 948–61.

54 Vermeer AMC, Lodder EM, Thomas D, *et al.* Dilation of the Aorta Ascendens Forms Part of the Clinical Spectrum of HCN4 Mutations. J. Am. Coll. Cardiol. 2016; **67**: 2313–5.

55 Guo DC, Gong L, Regalado ES, *et al.* MAT2A mutations predispose individuals to thoracic aortic aneurysms. *Am J Hum Genet* 2015; **96**: 170–7.

56 Combs MD, Knutsen RH, Broekelmann TJ, *et al.* Microfibril-associated glycoprotein 2 (MAGP2) loss of function has pleiotropic effectsin vivo. *J Biol Chem* 2013; **288**: 28869–80.

57 Barbier M, Gross MS, Aubart M, *et al.* MFAP5 loss-of-function mutations underscore the involvement of matrix alteration in the pathogenesis of familial thoracic aortic aneurysms and dissections. *Am J Hum Genet* 2014; **95**: 736–43.

58 Micha D, Guo DC, Hilhorst-Hofstee Y, *et al.* SMAD2 Mutations Are Associated with Arterial Aneurysms and Dissections. *Hum Mutat* 2015; **36**: 1145–9.

59 Schepers D, Tortora G, Morisaki H, *et al.* A mutation update on the LDS-associated genes TGFB2/3 and SMAD2/3. *Hum Mutat* 2018; **39**: 621–34.

60 Zhang W, Zeng Q, Xu Y, *et al.* Exome sequencing identified a novel SMAD2 mutation in a Chinese family with early onset aortic aneurysms. *Clin Chim Acta* 2017; **468**: 211–4.

61 Bertoli-Avella AM, Gillis E, Morisaki H, *et al.* Mutations in a TGF-β ligand, TGFB3, cause syndromic aortic aneurysms and dissections. *J Am Coll Cardiol* 2015; **65**: 1324–36.

62 Tan KL, Haelterman NA, Kwartler CS, *et al.* Ari-1 Regulates Myonuclear Organization Together with Parkin and Is Associated with Aortic Aneurysms. *Dev Cell* 2018; **45**: 226-244.e8.

63 Guo D chuan, Regalado ES, Pinard A, *et al.* LTBP3 Pathogenic Variants Predispose Individuals to Thoracic Aortic Aneurysms and Dissections. *Am J Hum Genet* 2018; **102**: 706–12.

64 Lemaire SA, McDonald MLN, Guo DC, *et al.* Genome-wide association study identifies a susceptibility locus for thoracic aortic aneurysms and aortic dissections spanning FBN1 at 15q21.1. *Nat Genet* 2011; **43**: 996–1002.

65 Guo D chuan, Hostetler EM, Fan Y, *et al.* Heritable Thoracic Aortic Disease Genes in Sporadic Aortic Dissection. J. Am. Coll. Cardiol. 2017; **70**: 2728–30.

66 Vasan RS, Glazer NL, Felix JF, *et al.* Genetic variants associated with cardiac structure and function: A meta-analysis and replication of genome-wide association data. *JAMA - J Am Med Assoc* 2009; **302**: 168–78.
